# Myopathic Lamin Mutations Cause Reductive Stress and Activate the Nrf2/Keap-1 Pathway

**DOI:** 10.1371/journal.pgen.1005231

**Published:** 2015-05-21

**Authors:** George Dialynas, Om K. Shrestha, Jessica M. Ponce, Monika Zwerger, Dylan A. Thiemann, Grant H. Young, Steven A. Moore, Liping Yu, Jan Lammerding, Lori L. Wallrath

**Affiliations:** 1 Department of Biochemistry, University of Iowa, Iowa City, Iowa, United States of America; 2 Interdisciplinary Graduate Program in Genetics, University of Iowa, Iowa City, Iowa, United States of America; 3 Weill Institute for Cell and Molecular Biology, Department of Biomedical Engineering, Cornell University, Ithaca, New York, United States of America; 4 Department of Pathology, University of Iowa, Iowa City, Iowa, United States of America; 5 NMR Facility, Carver College of Medicine, University of Iowa, Iowa City, Iowa, United States of America; Johns Hopkins University School of Medicine, UNITED STATES

## Abstract

Mutations in the human *LMNA* gene cause muscular dystrophy by mechanisms that are incompletely understood. The *LMNA* gene encodes A-type lamins, intermediate filaments that form a network underlying the inner nuclear membrane, providing structural support for the nucleus and organizing the genome. To better understand the pathogenesis caused by mutant lamins, we performed a structural and functional analysis on *LMNA* missense mutations identified in muscular dystrophy patients. These mutations perturb the tertiary structure of the conserved A-type lamin Ig-fold domain. To identify the effects of these structural perturbations on lamin function, we modeled these mutations in Drosophila *Lamin C* and expressed the mutant lamins in muscle. We found that the structural perturbations had minimal dominant effects on nuclear stiffness, suggesting that the muscle pathology was not accompanied by major structural disruption of the peripheral nuclear lamina. However, subtle alterations in the lamina network and subnuclear reorganization of lamins remain possible. Affected muscles had cytoplasmic aggregation of lamins and additional nuclear envelope proteins. Transcription profiling revealed upregulation of many Nrf2 target genes. Nrf2 is normally sequestered in the cytoplasm by Keap-1. Under oxidative stress Nrf2 dissociates from Keap-1, translocates into the nucleus, and activates gene expression. Unexpectedly, biochemical analyses revealed high levels of reducing agents, indicative of reductive stress. The accumulation of cytoplasmic lamin aggregates correlated with elevated levels of the autophagy adaptor p62/SQSTM1, which also binds Keap-1, abrogating Nrf2 cytoplasmic sequestration, allowing Nrf2 nuclear translocation and target gene activation. Elevated p62/SQSTM1 and nuclear enrichment of Nrf2 were identified in muscle biopsies from the corresponding muscular dystrophy patients, validating the disease relevance of our Drosophila model. Thus, novel connections were made between mutant lamins and the Nrf2 signaling pathway, suggesting new avenues of therapeutic intervention that include regulation of protein folding and metabolism, as well as maintenance of redox homoeostasis.

## Author Summary

Mutations in the human *LMNA* gene cause muscular dystrophy that is often accompanied by heart disease. The *LMNA* gene makes proteins that form a network on the inner side of the nuclear envelope, a structure that reinforces the cell nucleus. How mutations in the *LMNA* gene cause muscle disease is not well understood. Our studies provide evidence that *LMNA* mutations activate an intracellular signaling pathway and alter the redox homeostasis of muscle tissue. Thus, our results suggest that blocking the signaling pathway and maintaining the oxidative state of the diseased muscle are potential therapies for muscular dystrophy patients with *LMNA* mutations.

## Introduction

The human *LMNA* gene exemplifies the rich source of genetic variation that exists in the human genome. Over 283 sequence variants and 460 disease-causing mutations have been identified to date. These mutations cause at least 13 distinct clinical diseases, called laminopathies, which have mainly tissue-restricted phenotypes, despite the fact that A-type lamins are expressed in nearly all cells [1]. For any given disease, mutations are scattered throughout the *LMNA* gene [2]. Furthermore, neighboring missense mutations can give rise to dramatically different disease phenotypes. These findings suggest that defined protein domains do not have tissue-specific functions.

The *LMNA* gene encodes alternatively spliced mRNAs for lamin A and C that have a common domain structure [3]. The N-terminal region of lamins forms a globular domain, the central region forms a coiled coil domain, and the carboxy terminus contains an Ig-fold domain [4]. Lamins dimerize through the rod domain and form filaments via head-to-tail interactions of the dimers. Lateral interactions between lamin filaments are thought to generate higher order structures that form the network that underlies the inner membrane of the nuclear envelope. This network provides structural stability to the nucleus, serves as a scaffold for inner nuclear envelope proteins, and organizes the genome through contacts made with chromatin [5].

The mechanisms by which mutant lamins cause disease remain incompletely understood. It has been proposed that mutant lamins cause nuclear fragility, leading to nuclear deformation and breakage under mechanical stress [6]. This idea provides an explanation for the tissue-restricted phenotypes associated with muscular dystrophy and cardiomyopathy. However, sensitivity to mechanical stress does not explain why mutant lamins cause other diseases, such as lipodystrophy. For tissues that do not experience mechanical stress, mutant lamins are proposed to dysregulate gene expression [7]. While evidence exists for both the mechanical stress and gene expression models, it is also possible that lamins are required for adult stem cell homeostasis [8].

To gain novel insights into mechanisms by which mutant lamins cause disease, we previously developed a Drosophila model of lamin associated muscular dystrophy [9]. Mutations identified in patients are modeled into Drosophila Lamin C. Tissue-specific expression achieved by the Gal4/UAS system provides a means of expressing the mutant lamins in desired tissues [10]. Expression of the mutant lamins in larval body wall muscle causes larval locomotion defects and pupal death [9].

Here, we report in-depth structural and functional analyses of the mutant lamins identified in muscular dystrophy patients. Structural studies, which included NMR analysis, showed that the pathogenic mutations perturb the tertiary structure of the lamin Ig-fold domain. These structural perturbations are associated with cytoplasmic lamin aggregation, activation of the Nrf2/Keap-1 pathway, and reductive stress, yet have minimal effects on nuclear stiffness. These data lead to a novel hypothesis suggesting that cytoplasmic aggregation of nuclear envelope proteins causes Nrf2 target gene activation. Our findings provide new potential avenues for therapy involving protein metabolism and redox homeostasis.

## Results

### Mutant lamins alter the tertiary structure of the Ig-fold domain

To identify mechanisms by which *LMNA* mutations cause muscle disease, we performed an in-depth structural analysis on four mutations identified in patients with skeletal muscular dystrophy. Each patient possessed a single nucleotide substitution in *LMNA* that caused an amino acid substitution in the Ig-fold domain of A-type lamins. These amino acid substitutions (G449V, N456I, L489P and W514R) were dispersed throughout the Ig-fold domain and map to loop regions, making their effect on protein structure challenging to predict (S1 Fig). To analyze the effects of these amino acid substitutions on Ig-fold structure, sequences encoding the wild type and mutant human A-type lamin Ig-fold domain were cloned into an expression vector, expressed and purified from *E*. *coli* (S2A Fig). The peptides were analyzed by circular dichroism (CD) and NMR. The wild type A-type lamin Ig-fold domain contains eight anti-parallel and one parallel beta strands that form a beta barrel structure [11] (S1 Fig). The CD spectra for the wild type and three of the mutant Ig-fold domains showed similar peak intensity at 220 nm (S2B Fig) demonstrating that the beta-sheet content of the wild type and mutant Ig-fold domains was comparable.

Given the absence of obvious changes in beta sheet content between the wild type and mutant Ig-fold domains, we examined whether the amino acid substitutions altered the tertiary structure of the Ig-fold domain. Changes in tertiary structure often affect the thermal stability of a protein. We determined the T_1/2_ for denaturation of the wild type Ig-fold to be 55°C (S2C Fig), which was slightly lower than the published value of 62°C [11]. This variation might be accounted for by slight differences in the size of the domain in the expression constructs. Our construct included amino acid residues 435–552, whereas the published construct included amino acid residues 411–553 [11]. The T_1/2_ values for denaturation of G449V, N456I and W514R were reduced to 40, 35 and 35°C, respectively (S2C Fig). Thus, all three of the mutants analyzed had significantly lowered the thermal stability of the Ig-fold compared to that of the wild type Ig-fold domain.

**Fig 1:**
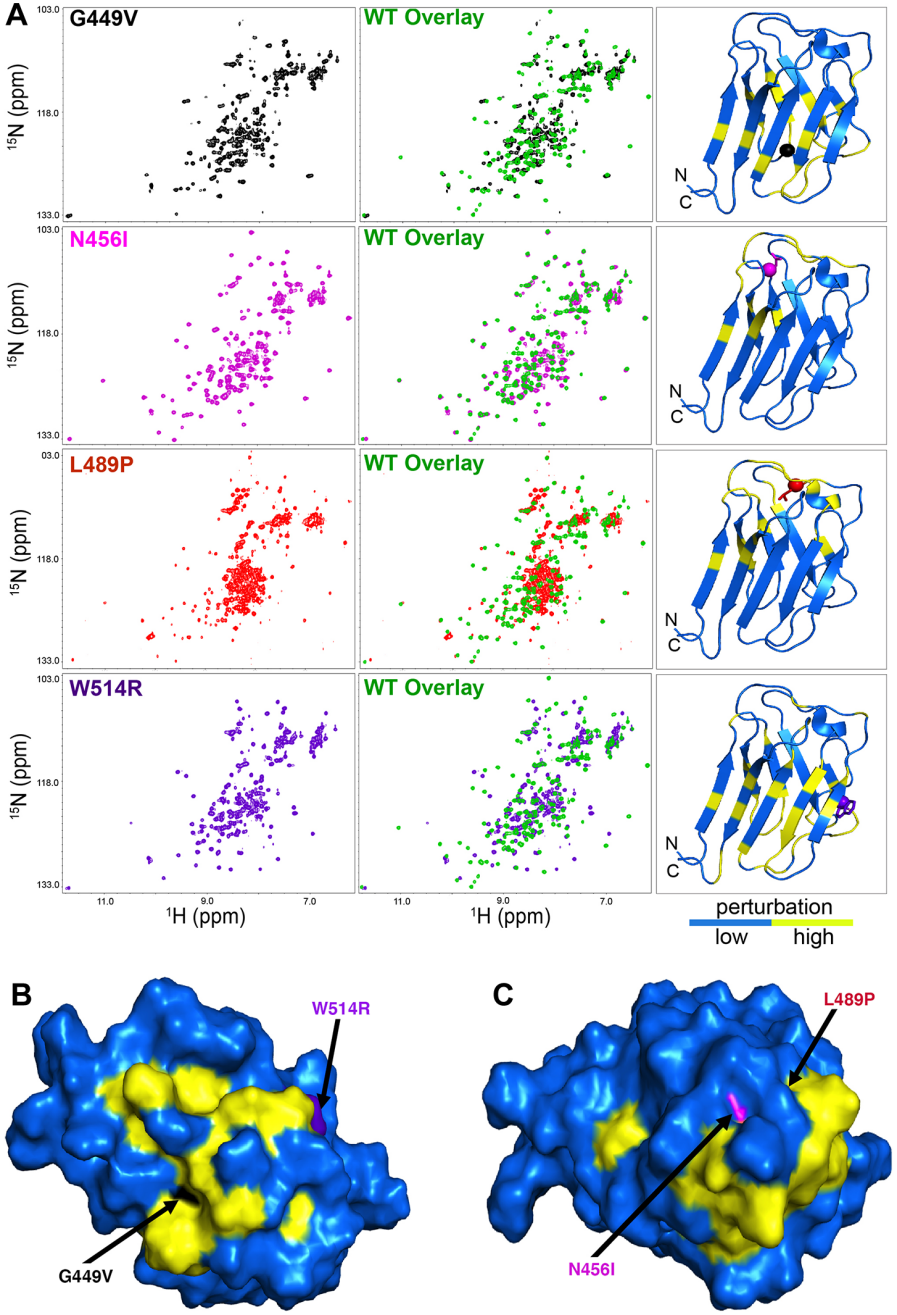
The *LMNA* mutations cause perturbations of the A-type lamin Ig-fold tertiary structure. (A) The ^15^N/^1^H HSQC NMR spectrum of each mutant Ig-fold (left column) was superimposed onto that of the wild type Ig-fold in green (middle column) PDB 1IVT was used to generate a ribbon plot of the wild type Ig-fold showing the perturbed amino acids (yellow, Δδ_ppm_ ≥ 0.15 ppm) and the unperturbed backbone atoms (blue, Δδ_ppm_ ≤ 0.15 ppm) backbone (right column). Perturbation was determined by calculating the chemical shift difference for each backbone amide cross peak using the equation Δδ_ppm_ = ([Δδ(^1^H_ppm_)]^2^ + [0.1 • Δδ(^15^N_ppm_)]^2^) ^1/2^. Since the ^15^N/^1^H HSQC spectrum of the wild type was assigned (S3B Fig) and the spectra of the mutants were not assigned, the Δδ_ppm_ was calculated by comparing each backbone amide cross peak in the wild type spectrum (assigned) to all cross peaks present in the mutant spectrum. The smallest Δδ_ppm_ from this calculation was taken as the chemical shift perturbation value for that residue. In the ribbon plots, the mutated residues are shown in stick mode with their C atoms indicated by spheres. (B and C) Surface display of the Ig-fold domain showing common residues perturbed by G449V and W514R (B) and N456I and L489P (C). Amino acids not perturbed are shown in blue. Amino acid residues altered by the *LMNA* mutations are colored by black (G449V), purple (W514R), magenta (N456I) and red (L489P) and indicated by an arrow. Note that L489P is buried and not visible from the surface.

In the absence of changes in secondary structure, a lower T_1/2_ value for thermal stability for the mutant proteins suggested structural perturbations (i.e. altered positioning of amino acids) within the Ig-fold domain tertiary structure. The ^15^N/^1^H Heteronuclear Single Quantum Coherence (HSQC) NMR spectrum of the wild type Ig-fold domain of our construct showed well dispersed cross peaks, similar to those reported, indicating that the wild type protein is well folded and has similar tertiary structures as reported previously [11] (S3A Fig). However, subtle differences are clearly observed between the two ^15^N/^1^H HSQC spectra, due to differences in the expression constructs used (see above). To determine whether the amino acid substitutions caused changes in the tertiary structure of the Ig-fold domain, we assigned the backbone amide cross peaks in the ^15^N/^1^H HSQC spectrum of the wild type Ig-fold (S3B Fig) and compared it to that generated from the ^15^N/^1^H HSQC spectrum of each mutant (Fig 1A). All mutants showed significant changes in the ^15^N/^1^H HSQC spectrum. In all cases, perturbations were observed in the loop in which the amino acid substitution occurred. In addition, G449V and W514R showed chemical shift perturbations throughout the beta sheets of the Ig-fold domain, indicating that these two mutations caused large structural changes. Among the four mutants tested, N456I exhibited a spectrum most similar to that of the wild type Ig-fold, while the L489P mutant showed a mixed population of folded and unfolded protein. Analysis of the chemical shift perturbation data revealed two clusters of commonly perturbed residues, one shared between G449V and W514R (Fig 1B) and other shared between N456I and L489P (Fig 1C). These commonly perturbed residues are on opposite sides of the Ig-fold barrel (Fig 1C). Taken together, our analysis identified two surfaces on the Ig-fold that are critical for the function of A-type lamins in muscle.

### Mutant lamins have minimal dominant effects on nuclear stiffness

Lamins A and C are contributors to nuclear stiffness [12,13]. Given the structural perturbations of the mutant Ig-fold domains, we tested whether full-length lamins possessing the amino acid substitutions within the Ig-fold altered nuclear deformation in response to mechanical stress. To accomplish this, we made use of a Drosophila model [9]. Drosophila Lamin C shows conservation of amino acid sequence and domain organization with human lamin A/C, including the amino acid residues under investigation [9]. Note that the amino acid numbering for the substituted residues is different between the human and Drosophila Ig-fold (S1 Fig) due to differences in the size of the domain between the two species. Importantly, the carboxyl sequence of Drosophila Lamin C is predicted to form an Ig-fold structure that is highly similar to that of human lamin A (S4 Fig). In addition, the spatial and temporal expression pattern of Drosophila Lamin C is conserved with that of human lamin A/C [14]. Mutations identified in human *LMNA* were modeled into the Drosophila *Lamin C* gene, transgenic Drosophila were generated and the Gal4/UAS system [10] was used to express wild type and mutant lamins in Drosophila larval body wall muscle at levels comparable to endogenous Lamin C [9].

**Fig 2:**
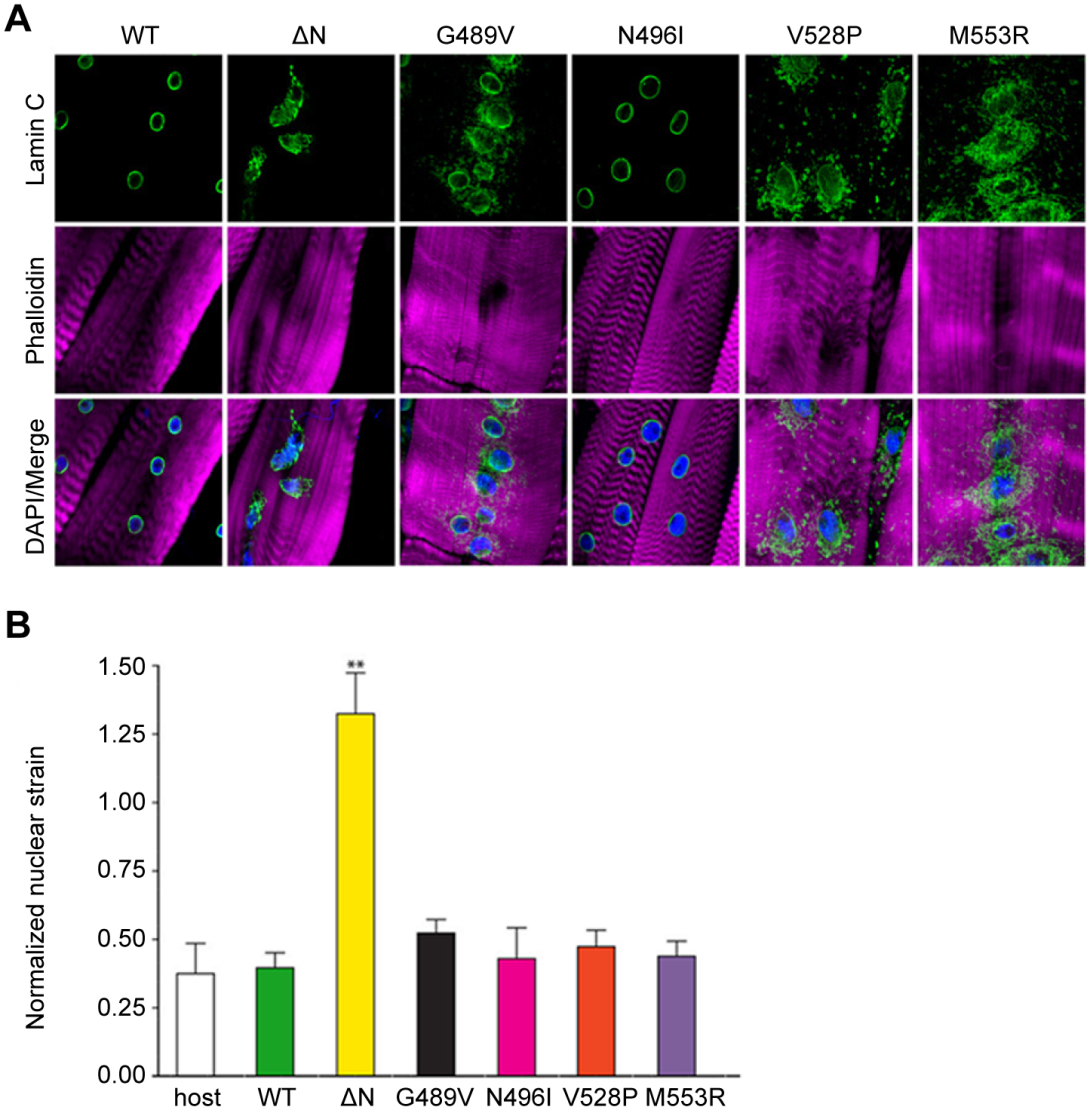
Mutant lamins mislocalize in Drosophila larval body wall muscles and have minimal dominant effects on nuclear stiffness. (A) Immunohistochemical staining of Drosophila body wall muscles from transgenic larvae expressing full length wild type (WT) and mutant Lamin C. Phalloidin staining is represented by magenta, Lamin C by green, and DAPI by blue. (B) Normalized nuclear strain values for nuclei in Drosophila larval body wall muscle. Eleven nuclei from three different larvae were analyzed per genotype. Error bars represent standard error of the mean. Increased normalized nuclear strain values correspond to decreased nuclear stiffness. The ** indicates p ≤ 0.01 compared to the value obtained for muscles expressing wild type Lamin C.

Expression of wild type Drosophila Lamin C in the larval body wall muscle caused no obvious phenotypes and did not affect viability [9]. In contrast, muscle-specific expression of mutant Lamin C caused larval locomotion defects and semi-lethality at the pupal stage [9]. Approximately 30% of the larval body wall muscles showed abnormally shaped and spaced nuclei, disorganization of the actin cytoskeleton, and cytoplasmic aggregation of nuclear envelope proteins including mutant Lamin C (Fig 2A) and nuclear pore proteins as shown previously [9]. None of these cellular phenotypes were observed in the wild type Lamin C control where lamin localization was confined to the nucleus (Fig 2A).

To examine the effect of mutant lamins on nuclear stability in muscle tissue, larval body wall muscle fillets were hand-dissected from transgenic Drosophila larvae and attached to a flexible silicone membrane for nuclear strain measurements [13]. Expression of wild type Lamin C in an otherwise wild type background did not alter nuclear stiffness relative to that of a non-transgenic stock (Fig 2B). In contrast, expression of Lamin C in which the N-terminal head domain had been deleted (ΔN) showed larger nuclear deformation (Fig 2A) corresponding to decreased nuclear stiffness (Fig 2B). These results are consistent with the dominant negative effects observed for headless lamin A in the cultured cells [13]. No statistical difference in nuclear tension was observed in myonuclei expressing wild type and mutant Lamin C. Thus, these show that the mutant lamins do not have dominant effects on the nuclear tension in Drosophila muscle fibers.

### Mutant lamins alter muscle gene expression

**Fig 3:**
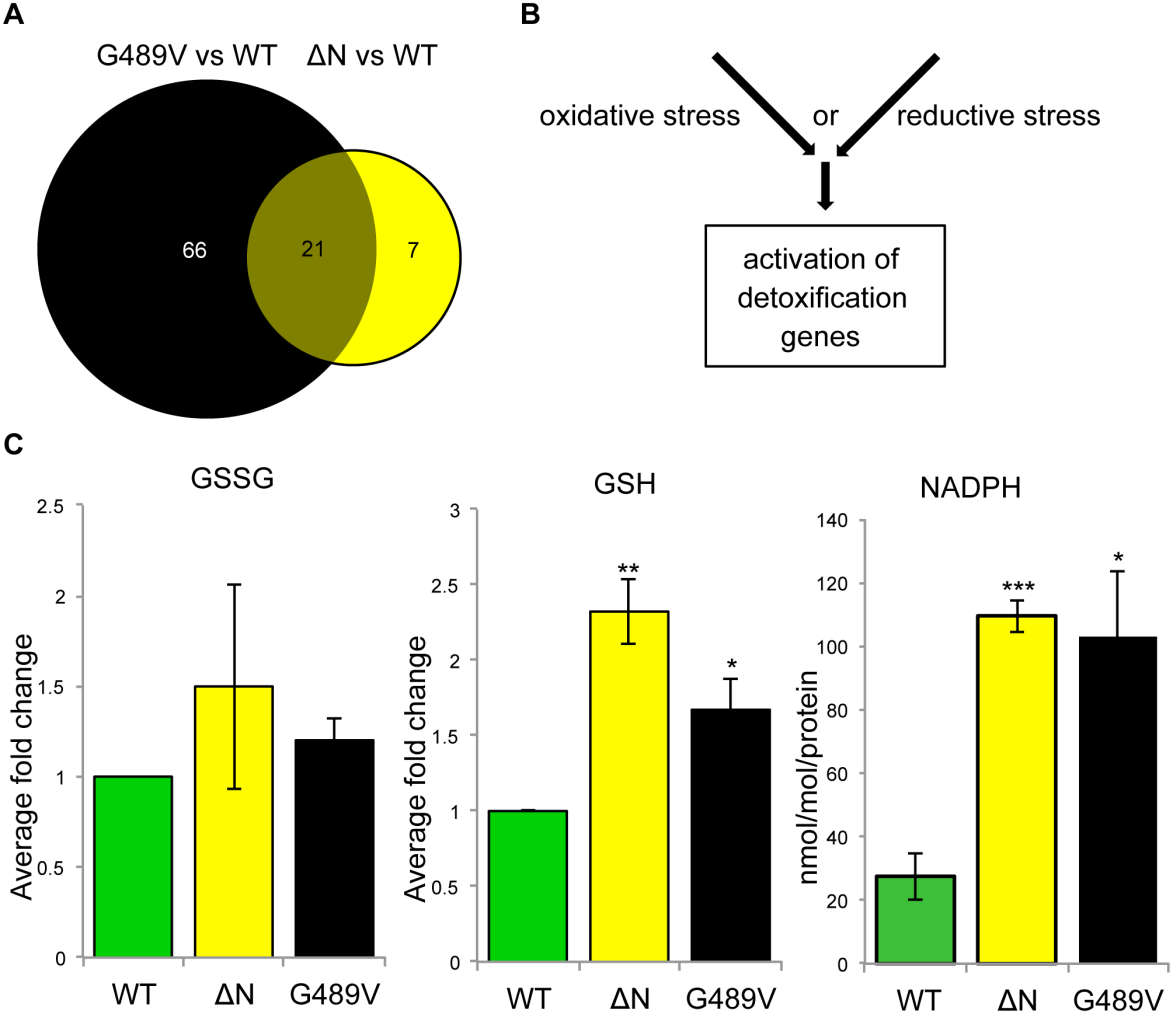
Mutant lamins cause changes in Drosophila muscle gene expression and reductive stress. (A) Venn diagram of the changes in gene expression caused by ΔN and G489V versus wild type Lamin C. (B) Alternative pathways known to cause activation of cellular detoxification genes. (C) Quantitation of oxidized glutathione (GSSG), reduced glutathione (GSH) and NADPH in Drosophila body wall muscles of larvae expressing wild type and mutant Lamin C. Analysis was performed on three independent biological samples. Error bars represent standard error of the mean. Statistical significance is indicated by * for p ≤ 0.05, ** for p ≤ 0.01, and *** for p ≤ 0.001 when compared to values obtained for muscles expressing wild type Lamin C.

In the absence of dominant changes in nuclear stiffness, we reasoned that mutant lamins might exert their pathogenic effects by changing muscle gene expression [15–17]. To minimize indirect effects on gene expression, we exploited the Drosophila model to capture changes in muscle gene expression 24–48 hours following induction of mutant *Lamin C* expression. Total RNA was isolated from larval body wall muscles and used for Affymetrix gene expression profiling. The analysis was performed on transgenic larvae expressing wild type, ΔN and G489V full-length versions of Lamin C. Lamin C ΔN was selected because it is known to have dominant negative effects on lamin assembly. Lamin C G489V was selected because it caused the greatest percent lethality (95%) [9]. Using the Partek software suite and a two-fold cut off with a p value of 0.05 or greater, 28 genes showed changes in expression between muscle expressing wild type Lamin C and ΔN (Fig 3A and S1 Table). A total of 87 genes showed changes in expression between wild type Lamin C and the G489V mutant (Fig 3A and S2 Table), with 21 genes overlapping with those altered by the ΔN mutant (Fig 3A and Table 1). The majority of these genes were up-regulated in response to the mutant lamins, consistent with a repressive role of wild type lamins in gene expression [15,16]. The relatively small number of genes that changed expression is consistent with the idea that ‘first responder’ genes were captured by the analysis.

**Table 1:** Genes misregulated by Lamin C ΔN and G489V.

Gene symbol	Gene name	WT vs ΔN		WT vs G489V		Function
		Fold change	p value	Fold change	p value	
*Cyp4p2*	*Cyp4p2*	144.40	7.53E-07	71.70	1.85E-06	Electron carrier activity; heme binding; iron ion binding; oxidoreductase activity*
*PlexB*	*Plexin B*	23.84	4.09E-08	34.56	2.11E-08	Axon guidance
*CG9935*	-	8.77	4.18E-06	3.48	1.03E-05	Glutamate gated ion channel activity*
*CG34007*	-	7.96	7.33E-05	33.49	3.47E-06	Unknown
*CG1074*	-	7.36	2.87E-07	2.14	8.15E-05	Methyltransferase activity; nucleic acid binding*
*CG31781*	-	5.50	5.18E-06	4.21	3.05E-04	Lateral inhibition in cell fate determination
*GstD9*	*Glutathione S transferase D9*	4.91	9.40E-05	8.66	1.63E-05	Glutathione transferase activity
*CG2064*	-	4.21	1.43E-04	5.88	4.35E-05	Oxidoreductase activity*
*CG32021*	-	3.39	3.25E-05	2.68	2.11E-04	Lateral inhibition in cell fate determination
*CG14906*	-	3.72	4.37E-06	4.34	2.27E-06	Methyltransferase activity; nucleic acid binding*
*CG33494*	-	3.60	9.57E-05	3.42	1.21E-04	Unknown
*Arc1*	*Activity-regulated cytoskeleton associated protein 1*	2.89	4.82E-05	3.07	3.47E-05	Nucleic acid binding; zinc ion binding*
*CG3448*	-	2.41	5.93E-05	6.14	8.68E-07	DNA binding*
*alphaTub84D*	*alpha-Tubulin at 84D*	2.39	7.62E-05	2.66	3.93E-05	GTP binding; GTPase binding; structural constituent of cytoskeleton*; Myosin binding
*RpS11*	*Ribosomal protein S11*	2.04	2.74E-05	2.01	3.06E-05	Structural constituent of ribosome*
*CG16787*	-	2.03	2.18E-05	3.01	1.48E-06	Unknown
*Sclp*	*Sclp*	-2.49	7.23E-06	-3.88	7.11E-07	Muscle function
*CG10365*	-	-2.54	1.77E-04	-4.20	1.51E-05	Unknown
*NnaD*	*Nna1 ortholog*	-2.56	4.34E-05	-2.46	5.55E-05	Metallocarboxypeptidase activity; purine nucleotide binding; zinc ion binding*; larval and neural retina development; mitochondrion organization
*l(2)03659*	*lethal(2)03659*	-5.08	1.47E-06	-4.60	2.13E-06	ATP binding; ATPase activity; transporter activity*
*gkt*	*glaikit*	-6.31	1.60E-07	-5.04	3.46E-07	3’-tyrosyl-DNA phosphodiesterase activity*; nervous system development; epithelial cell apical/basil polarity

*Inferred from amino acid sequence

Using Partek and Flybase gene annotations, we discovered that cellular detoxification genes, such as *glutathione S transferase* (*Gst*) genes, were enriched among those that changed expression (Table 2). These genes are typically activated in response to oxidative stress [18,19]. Other genes, including those involved in neuromuscular junction function (Table 2, S1 and S2 Tables) might be activated to compensate for deterioration of the muscles at the neuromuscular junction. Thus, the gene expression analysis provided insights on the initial stages of pathogenesis.

**Table 2:** Categories of mis-regulated genes differentially expressed in wild type Lamin C vs G489V.

Categories	Examples of genes	# Genes
Cellular detoxification	*Cyp4p2; GstD4; GstD9; Prx2540-2*	12
DNA/chromosome metabolism	*Ada2b; Arc1; NfI;RpA-70*	12
Neuromuscular junction	*Gkt; plexB; Sclp; Sh*	10
Other function	*Ilp5; l(2)efl; Prp31; RpS11*	31
Unknown function	*CG9836; CG11475; CG14673; CG34115*	22

### Mutant lamins cause reductive stress

Cellular anti-oxidant genes are typically activated in response to a redox imbalance. Measurements of the levels of oxidized (GSSG) and reduced (GSH) glutathione were determined in extracts from body wall muscles from larvae expressing wild type, ΔN and G489V Lamin C transgene. This revealed similar levels of GSSG among all genotypes (Fig 3C, left panel). In contrast, GSH levels were elevated in muscle expressing the mutant Lamin C relative to wild type (Fig 3C, middle panel). Elevated levels of GSH and NADPH are hallmarks of a condition known as ‘reductive stress’ [20]. We found that NADPH levels were also elevated in the muscle of the larvae expressing ΔN and G489V, relative to wild type Lamin C (Fig 3C, right panel). These findings demonstrate that mutant lamins cause reductive stress in muscle.

To identify the source of the reductive stress, we measured the activity of NADPH-producing enzymes in larval body wall muscle. We discovered that the activity of glucose-6-phosphate dehydrogenase (G6PDH) and 6-phosphogluconate dehydrogenase (6PGH) were similar between muscles expressing wild type and mutant Lamin C (S5 Fig, left and middle panel). In contrast, the activity of isocitrate dehydrogenase (IDH) was elevated in muscles expressing mutant lamins, compared to that of wild type (S5 Fig, right panel). Thus, the elevated IDH activity provides a potential explanation for the increased levels of NADPH.

### Mutant lamins activate the Nrf2/Keap-1 pathway

Genes involved in cellular detoxification, such as the *Gst* genes, are typically activated by the conserved Nuclear factor erythroid 2-related factor 2 (Nrf2)/Kelch-like ECH associated protein 1 (Keap-1) signaling pathway [21,22]. Under normal conditions, the antioxidant transcription factor Nrf2 is sequestered in the cytoplasm by Keap-1. Under conditions of oxidative stress, cysteine residues within Keap-1 are oxidized, causing Nrf2 to no longer associate and translocate into the nucleus, where it activates target genes possessing anti-oxidant response elements (AREs) [21,22]. However, an alternative mechanism for Nrf2 target gene activation has been described for conditions of reductive stress [23] (Fig 3B). This mechanism relies on the competition between Nrf2 and p62/SQSTM1, an autophagy cargo acceptor, for the binding of Keap-1. Increased levels of p62/SQSTM1 sequester Keap-1, allowing Nrf2 to translocate into the nucleus and activate target genes.

**Fig 4:**
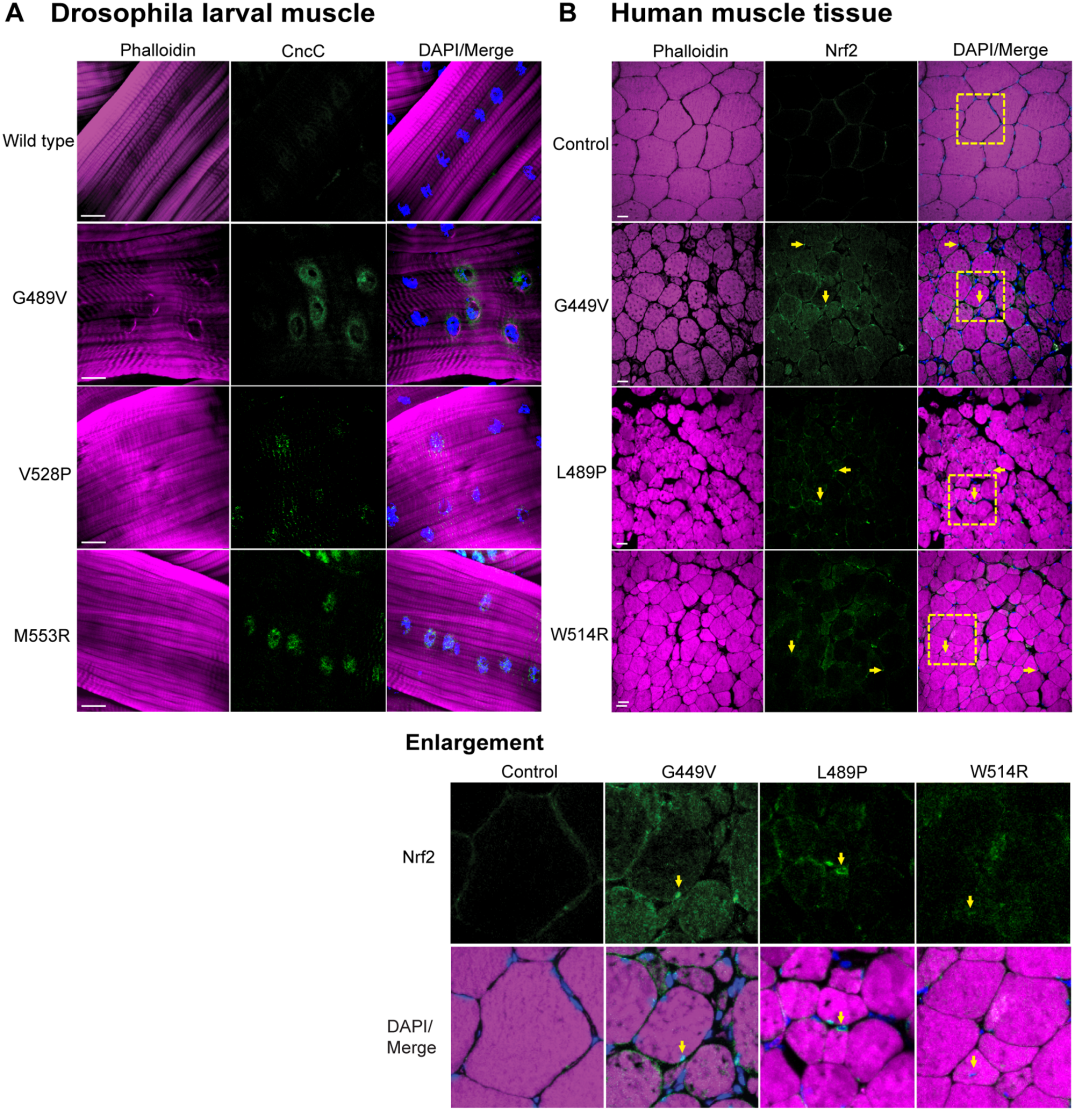
Mutant lamins cause enrichment of CncC/Nrf2 in myonuclei. (A) Drosophila larval body wall muscles from transgenic larvae stained with phalloidin (magenta), DAPI (blue) and CncC (green). Increased intensity of staining was observed for all mutants, relative to wild type. (B) Human muscle biopsy tissues were stained with phalloidin (magenta), DAPI (blue) and antibodies to Nrf2 (green). Arrows indicate myonuclei enriched for staining with Nrf2 antibodies. Boxed areas are those shown enlarged below.

Our finding that cellular detoxification genes were upregulated in response to mutant lamins suggested that the Nrf2/Keap-1 pathway was activated. To determine if this was the case, we performed immunohistochemistry on larval body wall muscle expressing wild type and mutant Lamin C with antibodies to Cap-and-collar C (CncC), the Drosophila homologue of Nrf2 [24]. In muscles expressing wild type Lamin C, we observed little to no staining, consistent with the fact that Nrf2/Keap-1 is rapidly turned over under normal conditions (Fig 4A) [25]. In contrast, we observed enhanced nuclear staining in muscles expressing each of the mutant Lamin C, relative to controls (Fig 4A). Thus, mutant lamins cause nuclear accumulation of CncC (Nrf2), which is consistent with activation of the Nrf2/Keap-1 pathway and expression of many CncC target genes.

To determine if the Nrf2/Keap-1 pathway is active in the human disease state, we stained muscle biopsy tissues from patients (possessing the mutations that were modeled in Drosophila) with antibodies to human Nrf2 [24]. In control muscle tissue, little to no staining was observed with the Nrf2 antibody (Fig 4B). In contrast, enhanced staining within the myonuclei of the patient muscle biopsy tissue was apparent (Fig 4B). Thus, expression of mutant lamins correlates with nuclear translocation of Nrf2 in both Drosophila and diseased human muscle.

**Fig 5:**
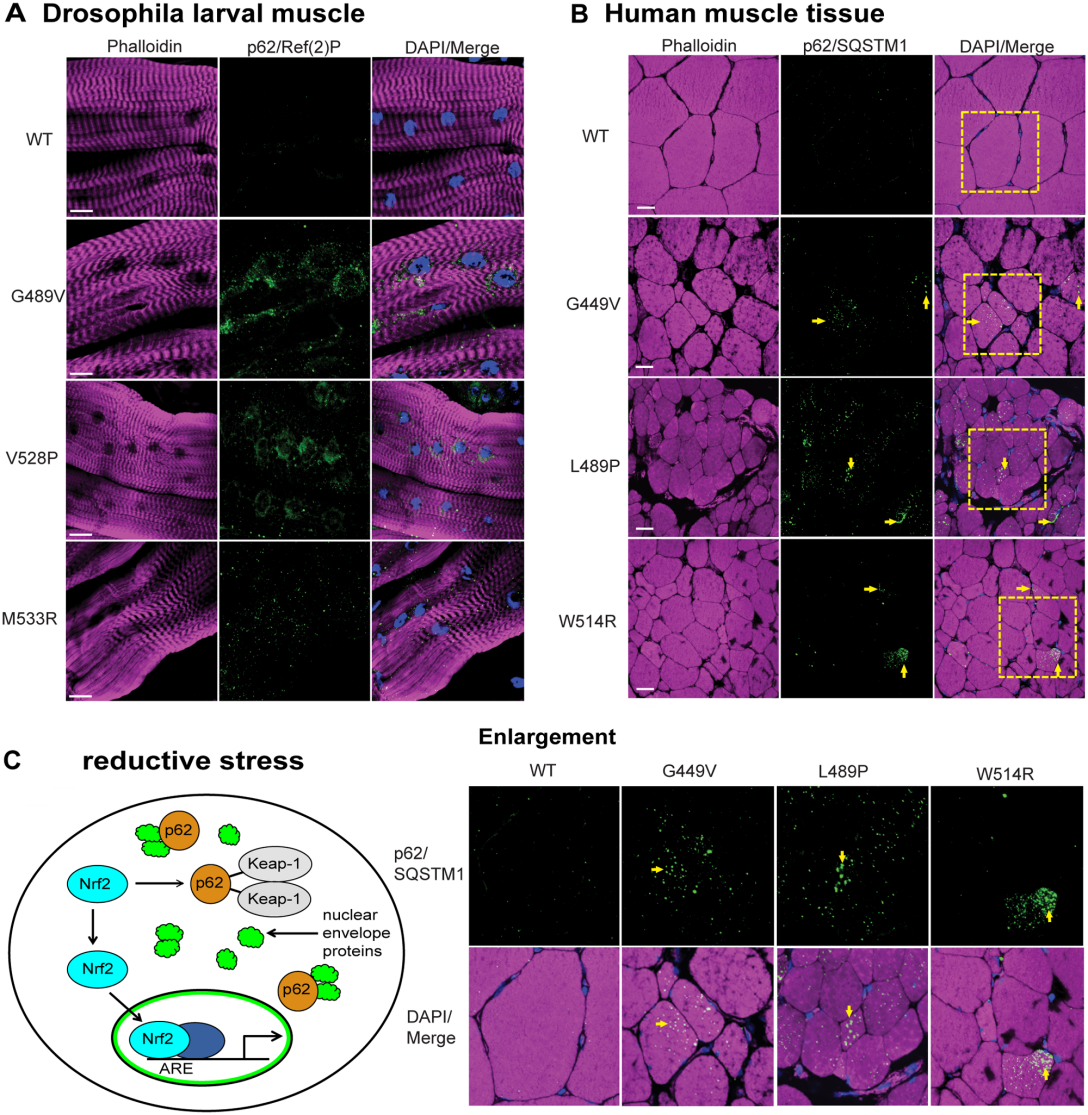
Mutant lamins cause increased levels of p62/SQSTM1 in Drosophila muscles and human muscle biopsy tissues. (A) Drosophila larval body wall muscles from transgenic larvae were stained with phalloidin (magenta), DAPI (blue) and antibodies to p62/Ref(2)P (green). Increased intensity of staining and the number of cytoplasmic foci were greater in muscle expressing mutant lamins compared to wild type. (B) Human muscle biopsy tissues were stained with phalloidin (magenta), DAPI (blue) and antibodies to p62/SQSTM1 (green). Arrows indicate muscle fibers with enhanced staining with anti-p62/SQSTM1 antibodies. Boxed areas are those shown enlarged below. (C) Unified model to explain the activation of cellular detoxification genes due to mutant lamins. Mutant lamins have altered tertiary structures that promote cytoplasmic aggregation. In addition, other nuclear envelope proteins such as LEM domain proteins and nuclear pore proteins accumulate in the cytoplasm via unknown mechanisms [9] (green cloud structures). These aggregates cause elevated levels of the autophagy adaptor protein p62/SQSTM1 (orange circles), which in turn binds Keap-1 (grey oval). This competitive binding allows Nrf2 (light blue oval) to translocate into the nucleus, bind to a partner protein (dark blue oval), and activate target genes possessing anti-oxidant response elements (AREs).

In mammalian systems Nrf2 and p62/SQSTM1 are co-regulated [21]. Given our findings of reductive stress and Nrf2 myonuclei enrichment, we hypothesized that levels of the autophagy cargo protein p62/SQSTM1 would be elevated in the muscles expressing mutant lamins relative to controls. To test this hypothesis, we stained Drosophila larval body wall muscles with antibodies to Drosophila p62/Ref(2)P, a homologue of human p62/SQSTM1 [26]. Muscles expressing wild type Lamin C showed hardly any staining for p62/Ref(2)P (Fig 5A). In contrast, muscles expressing mutant lamins showed increased cytoplasmic foci of staining (Fig 5A). The elevated levels of p62/Ref(2)P were validated by western analysis of protein extract from larval body wall muscles (S6 Fig). Thus, mutant lamins cause elevated levels of cytoplasmic p62/Ref(2)P, which is consistent with the increased cytoplasmic aggregation of mutant lamins (Fig 2A).

To determine if the elevated levels of p62/Ref(2)P also occur in the human disease state, we stained muscle biopsy samples from the patients (possessing the mutations that were modeled in Drosophila) with antibodies to human p62/SQSTM1 [26]. Scoring muscle fibers positive for p62/SQSTM1 if they had ten or more visible foci containing p62/SQSTM1, showed that the control patient muscle tissue had low levels of p62/SQSTM1; only 5/200 muscle fibers were positive (Fig 5B). In contrast, 48–62/200 fibers were scored positive in the patient samples. Furthermore, the diameter of the p62/SQSTM foci in the patient tissues was larger than those in the controls (Fig 5B). These findings strongly suggest that the Nrf2/Keap-1 pathway activation in both Drosophila and human muscle occurs through an alternative mechanism that is triggered by elevated levels of p62/SQSTM1.

## Discussion

Our structural studies of the lamin Ig-fold demonstrated that single amino acid substitutions in the loop regions perturb the tertiary structure, leaving the secondary structure of the folded domain largely intact (S2 Fig). These data were consistent with single-molecule force spectroscopy showing that the lamin Ig-fold possessing an R453W substitution required less force to unfold than the wild type Ig-fold domain [27]. Our structural data are consistent with *in silico* modeling in which amino acid substitutions in the Ig-fold that cause muscular dystrophy were predicted to alter the structure, more so than those that cause lipodystrophy or progeria [28]. Our NMR analysis of the mutant Ig-fold domains identified surfaces on opposite sides of the Ig-fold barrel that are critical for muscle function. This finding predicts that substitution of other amino acids that comprise these surfaces might result in muscular dystrophy. Consistent with this prediction, amino acid substitutions in eight of the 21 amino acids that make up these surfaces cause muscular dystrophy (Leiden muscular dystrophy database http://www.dmd.nl).

It is interesting to note that the largest structural perturbations were observed for the G449V and W514R mutants, which correspond to the most severe patient phenotypes. The corresponding amino acid substitutions in Drosophila Lamin C caused the greatest percentage of lethality [9]. The N456I mutant showed the least structural perturbations in the Ig-fold domain (Fig 1), though the relative severity of symptoms in this patient was not assertained [29]. Consistent with the structural data, the corresponding amino acid substitution in Drosophila Lamin C gave the least percentage of lethality [9]. Thus, our investigations showed an obvious correlation between the severity of the Ig-fold structural perturbations and phenotypic severity.

The structural perturbations within the Ig-fold might generate novel interaction surfaces that promote lamin aggregation (Figs 1 and 2A). Both nuclear and cytoplasmic aggregation of mutant lamins have been reported [30,31], however, they are not commonly observed in human muscle biopsy tissue or tissue from a laminopathy mouse model [9,32]. Cytoplasmic aggregation was observed for a truncated form of A-type lamin that causes Hutchinson-Gilford progeria syndrome [30]. Lamin aggregation is supported by X-ray crystallography studies of a R482W substitition in the A-type lamin Ig-fold domain that causes lipodystrophy [33]. The R482W Ig-fold domain possesses unique interaction surfaces not present in the wild type Ig-fold that form a unique platform for tetramerization.

The structural perturbations in the Ig-fold domain are likely to affect many functions of the mutant lamins. The lamin Ig-fold domain interacts with many partners to build the network that underlies the inner membrane of the nuclear envelope [5]. Mutant lamins can be inappropriately incorporated into the lamin network and function as dominant negatives [15,34,35]. This is the case for the headless lamin, which has dominant effects on nuclear shape and stiffness in both Drosophila muscle tissue and MEFs (Fig 2) [13]. In contrast, the Ig-fold substitutions do not cause major dominant effects on stiffness, however, there might be undetected alterations in the lamina and/or nuclear organization. Changes in nuclear organization could explain the misregulation of gene expression that we observed in muscles expressing mutant lamins (S1 and S2 Tables). In addition, the amino acid substitutions within the lamin Ig-fold domain might disrupt posttranslational modifications that occur on lamins, similar to what has been shown for familial partial lipodystrophy *LMNA* mutations that disrupt SUMOylation of Lamin A [36]. Changes in posttranslational modifications have the potential to alter interaction with partner proteins and/or affect aggregation properties.

Cytoplasmic protein aggregation has been linked to reductive stress [37,38]. Here, we show that cytoplasmic lamin aggregation correlates with elevated levels of both GSH and NADPH, hallmarks of reductive stress [20] (Fig 3). Elevated levels of isocitrate dehydrogenase enzyme activity (S5 Fig) contribute to the additional NADPH. In a similar manner, dominant negative forms of alphaB-crystallin (CryAB) result in cytoplasmic CryAB misfolding/aggregation and reductive stress in the mouse heart, ultimately leading to dilated cardiomyopathy [39]. These findings suggest that reductive stress might contribute to the dilated cardiomyopathy in cases of lamin associated muscular dystrophy. Interestingly, mutations in the human *CRYAB* gene cause disease phenotypes that are strikingly similar to those observed for lamin associated muscular dystrophy, including skeletal muscle weakness and dilated cardiomyopathy in cases of lamin-associated muscular dystrophy. It is worthwhile to note that CryAB functions as a chaperone to prevent aggregation of intermediate filament proteins such as desmin, suggesting a common link between intermediate filament aggregation and reductive stress.

An imbalance in redox homeostasis can provide an environment that promotes protein misfolding and aggregation. The redox state influences aggregation of lamins; aggregation has been observed under both oxidative and reductive conditions [33,40]. In fact, the formation of the novel tetramer generated by the R482W mutant Ig-fold domain (see above) required a reductive environment [33]. Reductive stress has also been observed in healthy individuals predisposed to Alzheimer disease, a disease of protein aggregation [41]. Alzheimer disease is typically accompanied by oxidative stress, however, lymphocytes from patients carrying an *ApoE4* allele that predisposes them to Alzheimer disease show reductive stress. It is hypothesized that continual activation of antioxidant defense systems, such as Nrf2/Keap-1 signaling, becomes exhausted over time, particularly later in life, resulting in the inability to properly defend against oxidative stress. Our redox analysis in Drosophila muscle occurred 24–48 hours post expression of the mutant lamins. Our findings suggest reductive stress at the onset of pathology that could resolve into oxidative stress later in disease progression [42].

Typically lamins are thought to regulate gene expression from inside the nucleus, by interacting with transcription factors and organizing the genome [5,43]. Our data support a novel model in which genes are misregulated as a consequence of mutant lamin aggregation in the cytoplasm. Cytoplasmic lamin aggregates have been found in high molecular weight complexes in cases of liver injury [44]. Such complexes contain nuclear pore proteins, signaling mediators, transcription factors and ribosomal proteins, which are thought to disrupt the normal cellular physiology. Lamin aggregation might also serve a cytoprotective function by facilitating the coalescence of mutant lamin so that the contractile apparatus can properly function. A similar mechanism exists in Huntington’s disease, where sequestration of mutant huntingtin in inclusion bodies correlates with better neuron health [45].

Collectively, our findings continue to support this Drosophila model of laminopathies, as many of the phenotypes discovered here in Drosophila have been validated in human muscle biopsies (Figs 4B and 5B) [9]. It is now possible to use this rapid genetic model to (1) determine if mutations in other domains of lamin produce similar phenotypes and (2) if lamin mutations have similar effects in other tissues, such as the heart. Our data suggest that cytoplasmic lamin aggregation contributes to muscle pathology. Consistent with this idea, increased rates of autophagy suppress phenotypes caused by mutant A-type lamin in cultured cells and mouse models [32,46]. Furthermore, electron microscopy of skeletal muscle biopsies from patients with *LMNA* mutations showed large perinuclear autophagosomes [47], similar to the localization of lamin aggregates and p62 foci in the Drosophila muscle (Figs 2A and 5A). Thus, the regulation of autophagy, a process that removes both damaged organelles and proteins, might be central to the development of therapies. The Drosophila model will allow for genetic dissection of both the autophagy and reductive stress pathways to identify the key factors responsible for the muscle pathogenesis and its suppression.

## Materials and Methods

### Protein expression

*LMNA* mutations identified in patients were introduced into a wild type copy of the human *LMNA* gene in a pCR2.1 vector via site-directed mutagenesis (Quick Change, Stratagene). The sequences of the PCR primers used to make these mutations are listed in S3 Table. DNA fragments encoding amino acids 435 through 552 of human lamin A were amplified using primers containing BamH1 and HindIII sites. The resulting PCR products were cloned into the pQE-30 Xa vector (Qiagen) and the constructs was expressed in M15[pREP4] *E*. *coli* cells. Expression was induced by IPTG overnight. Expression of wild type Ig-fold, G449V and W514R yielded protein that was purified by nickel column chromatography followed by Superdex-75 size exclusion chromatography. Expression of N456I and L489P yielded proteins that resided within inclusion bodies. Subsequent purification required denaturing conditions in 8 M urea during purification nickel and Superdex-200 size exclusion chromatography. Material from the monomeric peak eluted from the Superdex-200 column was dialyzed overnight to eliminate the urea and then re-purified on the Superdex-75 size exclusion column. Approximately 30 mg of wild type Ig-fold domain and approximately 8 mg of each mutant were purified per liter of cell culture, as determined spectrophotometrically (Nanodrop, Thermo Scientific).

### Biophysical analyses

Circular dichroism (CD) data was collected using 1 μM protein in 20 mM phosphate buffer with 100 mM NaCl and 0.1 mM DTT using a Jasco J815 CD Spectrophotometer. The spectral scan was performed between 190 nm and 280 nm. For T_1/2_ determination, melting curves were monitored under tryptophan absorbance at 230 nm; samples were heated at the rate of 2°C per minute. All CD experiments were performed in triplicate with independently prepared protein samples. Nuclear magnetic resonance (NMR) spectra were recorded at 20°C on a Bruker 500 or 800 MHz NMR spectrometer (NMR Core Facility, University of Iowa). NMR data were processed using NMRPipe [48] and analyzed using Sparky [49] and/or NMRView [50]. For the wild type Ig-fold domain, ^1^H, ^15^N, and ^13^C resonances of the backbone were assigned using the triple resonance experiments [HNCA, HN(CO)CA, HNCACB, HN(CO)CACB, HNCO, HN(CA)CO, and C(CO)NH-TOCSY] with a 500 uM ^15^N/^13^C-labeled sample. All NMR experiments were conducted in a NMR buffer containing 20 mM sodium phosphate (pH 7.0), 100 mM NaCl, 2 mM DTT, 1 mM EDTA and 0.1 mM sodium azide.

### Drosophila stocks

Drosophila stocks were cultured on standard corn meal media at 25°C [51]. Stocks with the wild type and mutant lamin transgenes were previously described [9,15,52]. All lamins were expressed using the Gal4/UAS system and the C57 muscle-specific Gal4 driver stock [10].

### Western analysis

Western analysis of protein extract from MEFs was according to published procedures [13]. Western analysis using Drosophila muscle was performed by extracting protein from 10 muscle fillets hand-dissected from third instar larvae in 2X Laemmli grinding buffer (125 mM Tris HCL, pH 6.8, 20% glucerol, 4% SDS, 0.005% bromophenol blue) plus 10 mM DL-Dithiothreitol. Anti-Drosophila p62 (1:8,000; kind gift of G. Juhász) and anti-Drosophila tubulin (1:300,000, Sigma) were used as primary antibodies and detected with anti-rabbit-HRP (1:400, Sigma) and anti-mouse-HRP (1:400, Sigma).

### Nuclear strain assays

Nuclear strain analysis of modified and unmodified MEFs was performed as previously described [53]. Nuclear strain analysis of Drosophila muscle was previously described [13].

### Gene expression analyses

Total RNA was isolated from hand-dissected body wall muscle from 40 third instar larvae per sample. RNA was purified using Trizol (Ambion) followed by RNAeasy (Qiagen). The RNA was used to generate labeled cRNA and hybridized to Drosophila 2.0 GeneChip arrays (Affymetrix) (DNA Core Facility, University of Iowa). Triplicate biological samples were analyzed for each genotype. The microarray data were analyzed using the Partek Genomic Suite [54]. Differentially expressed genes were identified using analysis of variance (ANOVA) with a two-fold change in expression and a *P* value of 0.05 or higher used as a cut off.

### Immunohistochemistry

Immunohistochemistry of Drosophila larval body wall muscles and human muscle biopsy tissues were performed as previously described [9]. Drosophila larval body wall muscles were stained with affinity purified anti-CncC antibodies (1:100; gift from H. Deng and T. Kerppola) [24], anti-p62/Ref(2)P (1:3,000, kind gift of G. Juhász) [55] that was detected with Alexa Fluor 488 goat anti-rabbit (1:400 dilution; Invitrogen). Filamentous actin was detected with Texas Red Phalloidin (1:400, Invitrogen). Human muscle biopsy cryosections were obtained from the Iowa Wellstone Muscular Dystrophy Cooperative Research Center and stained according to published procedures [9] with human p62/SQSTM1 (1:3,000, Sigma) and Nrf2 (1:300, Santa Cruz Biotech), followed by Alexa Fluor 488 goat anti-rabbit (1:400, Invitrogen). Filamentous actin was detected with Texas Red Phalloidin (1:400, Invitrogen).

### Glutathione and NADPH measurements

Drosophila larval body wall muscles were hand-dissected from 15 larvae, placed in 200 μl of 5% 5-sulfosalicylic acid and quantitation of reduced glutathione (GSH) and oxidized glutathione disulfide (GSSG) was performed as published [56]. GSSG was determined by adding a 1:1 mixture of 2-vinylpyridine and ethanol to the samples and incubating for two hours before assaying as described previously [57]. Enzymatic rates were compared to standard curves obtained from control samples. GSH and GSSG amounts were normalized to the protein content of the insoluble pellet from the 5-sulfosalicylic acid treatment, dissolved in 2.5% SDS in 0.1N bicarbonate, using the BCA Protein Assay Kit (Thermo Scientific). For measurements of NADPH, larval body wall muscles were hand-dissected from 15 larvae and immediately frozen in liquid nitrogen. Muscle samples were thawed in 170 μl of buffer [100 mM Tris HCl, 10 mM EDTA, 0.05% Triton X (v/v), pH 7.6] and sonicated four times for 30 seconds each. Assays were performed according to previous published procedures [58]. Absorbance was read at 310nm using a DU670 Spectrophotometer (Beckman) and enzyme activity was expressed as micromoles of NADPH per milligram of total protein.

### Enzymatic activity measurements

For measurements of the NADPH-producing enzymes, larval body wall muscles were hand-dissected from 15 larvae and immediately frozen in liquid nitrogen. For assays of G6PD and 6PGD, diethylenetriaminepentaacetic acid (DETAPAC) was added to pellet and the mixture sonicated using a Sonics Vibra-cell sonicator with a cup horn at 20% amplitude. Enzymatic assays were performed according to previous published procedures [59] using 0.1 M Tris HCl-MgCl_2_, 2mM NADP with either 0.034 grams of glucose-6-phosphate or 0.041 grams 6-phosphogluconic acid, pH 8.0. Absorbance was read at 340 nm using a DU670 Spectrophotometer (Beckman) and enzyme activity was reported as milliunits per milligram of protein. IDH activity was measured according to published procedures [60] using 100mM Tris-HCl, 0.10 mM NADP, 0.84 mM MgSO_4_, and1.37 mM isocitrate (pH 8.6). Absorbance was measured at 340 nm, every 9 sec, over 3 minutes at 25°C using a DU670 Spectrophotometer (Beckman) and enzyme activity was expressed as micromoles of NADPH produced per minute per microgram soluble protein X 10,000.

## Supporting Information

S1 Fig The positions of the amino acid substitutions in the human lamin A/C Ig-fold domain. Ribbon plot of the Ig-fold domain of human lamin A/C (PDB 1IVT). Amino acid residues altered by mutations in the human *LMNA* gene are indicated. The corresponding amino acid substitutions in Drosophila Lamin C are indicated in parentheses.
(TIF)

S2 Fig Muscle disease-causing amino acid substitutions alter the thermal stability and tertiary structure of the human lamin A/C Ig-fold domain. (A) Analysis of the purified wild type human lamin A/C Ig-fold domain using SDS PAGE following nickel column chromatography. Molecular weight markers are in lane 1. The flow through (FT), first wash (Wash-1), second wash (Wash-2) and elute (Elution) fractions are shown in lanes 2–5. The nickel affinity purified protein migrates to the anticipated molecular weight of the wild type Ig-fold, ~16 kDa. (B) CD spectra for the wild type and mutant Ig-fold domains expressed and purified from *E*. *coli*. The wild type and mutant Ig-fold domains possess beta sheet content as indicated by the peaks at 220 nm. The W514R substitution shows the absence of the peak at 232 nm due to the replacement of tryptophan. The majority of the L489P protein were unfolded as shown by the NMR data, therefore, CD data were not collected on this mutant. (C) Melting curves of wild type and mutant Ig-fold domains as determined by CD analysis at different temperatures. The T_1/2_, an indicator of thermal stability, was determined by calculating the midpoint of the curve between the start and end of the slope and are reported in the text.
(TIF)

S3 Fig NMR assignments of the ^15^N/^1^H HSQC spectrum of the wild type human lamin A/C Ig-fold domain of the construct used in this study. (A) Overlay of ^15^N/^1^H HSQC spectra of our Ig-fold domain construct (residues 435–552, green) and the reported Ig-fold construct (residues 428–549, reconstructed from BMRB5224, orange). The spectra are very similar indicating that they have similar tertiary structures. Subtle differences between the spectra are likely due to slight differences in the constructs used (see text). Due to these subtle differences, we performed backbone assignments of the amide cross peaks in order to unambiguously interpret the chemical shift perturbation data upon mutation. (B) Assigned ^15^N/^1^H HSQC spectrum of the Ig-fold domain of our wild type Ig-fold construct. The cross peaks were assigned by collecting and analyzing a suite of triple resonance NMR experiments using a ^15^N, ^13^C-labeled sample.
(TIF)

S4 Fig The human lamin A/C and Drosophila Lamin C Ig-fold have a conserved structure. The protein fold prediction program (HHpred-homology) was used to generate a predicted structure for the Ig-fold sequence of Drosophila Lamin C (green). This predicted structure was compared to that of the known structure of the human lamin A/C Ig-fold (PDB 1IVT) (blue).
(TIF)

S5 Fig Mutant lamins cause elevated levels of IDH. Quantitation of the activity of NADPH-producing enzymes, G6PD, 6PGD and IDH in Drosophila body wall muscles of larvae expressing mutant and wild type Lamin C. Analysis was performed on three independent biological samples. Error bars represent standard error of the mean. Statistical significance is indicated by * for p ≤ 0.05 when compared to values obtained for muscles expressing wild type Lamin C.
(TIF)

S6 Fig Mutant lamins cause increased levels of p62. (A) Western analysis of total protein isolated from body wall muscle showing increased levels of p62 in larvae expressing mutant lamin relative to the wild type control. An antibody to alpha tubulin was used as a loading control. (B) Graphical representation of the data obtained three westerns performed on independent biological samples is shown below. Error bars represent standard error of the mean. The * indicates p ≤ 0.05 when compared to the value obtained for wild type Lamin C.
(TIF)

S1 Table Changes in gene expression between wild type Lamin C and ΔN. Microarray analysis was performed using RNA isolated from muscle of third instar larvae expressing wild type Lamin C and Lamin C ΔN. The Partek software suite was used to identify genes that changes expression two-fold or greater with a p value of 0.05 or greater.
(DOCX)

S2 Table Changes in gene expression between wild type Lamin C and G489V. Microarray analysis was performed using RNA isolated from muscle of third instar larvae expressing wild type Lamin C and Lamin C G489V. The Partek software suite was used to identify genes that changes expression two-fold or greater with a p value of 0.05 or greater.
(DOCX)

S3 Table Lamin C mutagenesis primers. Primers were used to perform site-directed mutagenesis on a plasmid containing wild type Lamin C.
(DOCX)

## References

[1] Worman HJ (2012) Nuclear lamins and laminopathies. The Journal of Pathology 226: 316–325. 10.1002/path.2999 21953297 PMC6673656

[2] Worman HJ, Bonne G (2007) "Laminopathies": a wide spectrum of human diseases. Exp Cell Res 313: 2121–2133. 17467691 10.1016/j.yexcr.2007.03.028 PMC2964355

[3] Worman HJ, Fong LG, Muchir A, Young SG (2009) Laminopathies and the long strange trip from basic cell biology to therapy. J Clin Invest 119: 1825–1836. 10.1172/JCI37679 19587457 PMC2701866

[4] Herrmann H, Bar H, Kreplak L, Strelkov SV, Aebi U (2007) Intermediate filaments: from cell architecture to nanomechanics. Nat Rev Mol Cell Biol 8: 562–573. 17551517 10.1038/nrm2197

[5] Wilson KL, Berk JM (2010) The nuclear envelope at a glance. J Cell Science 123: 1973–1978. 10.1242/jcs.019042 20519579 PMC2880010

[6] Davidson PM, Lammerding J (2014) Broken nuclei—lamins, nuclear mechanics, and disease. Trends Cell Biol 24: 247–256. 10.1016/j.tcb.2013.11.004 24309562 PMC3972295

[7] Worman HJ, Courvalin JC (2004) How do mutations in lamins A and C cause disease? The Journal of Clinical Investigation 113: 349–351. 14755330 10.1172/JCI20832 PMC324546

[8] Gesson K, Vidak S, Foisner R (2014) Lamina-associated polypeptide (LAP)2alpha and nucleoplasmic lamins in adult stem cell regulation and disease. Semin Cell Dev Biol 29: 116–124. 10.1016/j.semcdb.2013.12.009 24374133 PMC4053830

[9] Dialynas G, Flannery KM, Zirbel LN, Nagy PL, Mathews KD, Moore SA, et al (2012) *LMNA* variants cause cytoplasmic distribution of nuclear pore proteins in Drosophila and human muscle. Hum Mol Genet 21: 1544–1556. 10.1093/hmg/ddr592 22186027 PMC3298278

[10] Duffy JB (2002) GAL4 system in Drosophila: a fly geneticist's Swiss army knife. Genesis 34: 1–15. 12324939 10.1002/gene.10150

[11] Krimm I, Ostlund C, Gilquin B, Couprie J, Hossenlopp P, Mornon JP, et al (2002) The Ig-like structure of the C-terminal domain of lamin A/C, mutated in muscular dystrophies, cardiomyopathy, and partial lipodystrophy. Structure 10: 811–823. 12057196 10.1016/s0969-2126(02)00777-3

[12] Lammerding J, Fong LG, Ji JY, Reue K, Stewart CL, Young SG, et al (2006) Lamins A and C but not lamin B1 regulate nuclear mechanics. J Biol Chem 281: 25768–25780. 16825190 10.1074/jbc.M513511200

[13] Zwerger M, Jaalouk DE, Lombardi ML, Isermann P, Mauermann M, Dialynas G, et al (2013) Myopathic lamin mutations impair nuclear stability in cells and tissue and disrupt nucleo-cytoskeletal coupling. Hum Mol Genet 22: 2335–2349. 10.1093/hmg/ddt079 23427149 PMC3658163

[14] Riemer D, Stuurman N, Berrios M, Hunter C, Fisher PA, Weber K, et al (1995) Expression of Drosophila Lamin-C Is Developmentally-Regulated—Analogies with Vertebrate a-Type Lamins. J Cell Science 108: 3189–3198. 7593280 10.1242/jcs.108.10.3189

[15] Dialynas G, Speese S, Budnik V, Geyer PK, Wallrath LL (2010) The role of Drosophila Lamin C in muscle function and gene expression. Development 137: 3067–3077. 10.1242/dev.048231 20702563 PMC2926956

[16] Kind J, van Steensel B (2010) Genome-nuclear lamina interactions and gene regulation. Curr Opin Cell Biol 22: 320–325. 10.1016/j.ceb.2010.04.002 20444586

[17] Mattout A, Pike BL, Towbin BD, Bank EM, Gonzalez-Sandoval A, Stadler MB, et al (2011) An EDMD mutation in C. elegans lamin blocks muscle-specific gene relocation and compromises muscle integrity. Curr Biol: CB 21: 1603–1614. 10.1016/j.cub.2011.08.030 21962710

[18] Saisawang C, Wongsantichon J, Ketterman AJ (2012) A preliminary characterization of the cytosolic glutathione transferase proteome from *Drosophila melanogaster* . Biochem J 442: 181–190. 10.1042/BJ20111747 22082028

[19] Zhou S, Campbell TG, Stone EA, Mackay TF, Anholt RR (2012) Phenotypic plasticity of the Drosophila transcriptome. PLoS Genet 8: e1002593 10.1371/journal.pgen.1002593 22479193 PMC3315458

[20] Brewer AC, Mustafi SB, Murray TV, Rajasekaran NS, Benjamin IJ (2013) Reductive stress linked to small HSPs, G6PD, and Nrf2 pathways in heart disease. Antioxid Redox Signal 18: 1114–1127. 10.1089/ars.2012.4914 22938199 PMC3567781

[21] Jain A, Lamark T, Sjottem E, Larsen KB, Awuh JA, Overvatn A, et al (2010) p62/SQSTM1 is a target gene for transcription factor NRF2 and creates a positive feedback loop by inducing antioxidant response element-driven gene transcription. Journal Biol Chem 285: 22576–22591. 10.1074/jbc.M110.118976 20452972 PMC2903417

[22] Nezis IP, Simonsen A, Sagona AP, Finley K, Gaumer S, Contamine D, et al (2008) Ref(2)P, the Drosophila melanogaster homologue of mammalian p62, is required for the formation of protein aggregates in adult brain. J Cell Biol 180: 1065–1071. 10.1083/jcb.200711108 18347073 PMC2290837

[23] Komatsu M, Kurokawa H, Waguri S, Taguchi K, Kobayashi A, Ichimura Y, et al (2010) The selective autophagy substrate p62 activates the stress responsive transcription factor Nrf2 through inactivation of Keap1. Nat Cell Biol 12: 213–223. 10.1038/ncb2021 20173742

[24] Deng H, Kerppola TK (2013) Regulation of Drosophila metamorphosis by xenobiotic response regulators. PLoS Genet 9: e1003263 10.1371/journal.pgen.1003263 23408904 PMC3567155

[25] Hayes JD, Dinkova-Kostova AT (2014) The Nrf2 regulatory network provides an interface between redox and intermediary metabolism. Trends Biochem Sci 39: 199–218. 10.1016/j.tibs.2014.02.002 24647116

[26] Bartlett BJ, Isakson P, Lewerenz J, Sanchez H, Kotzebue RW, Cumming RC, et al (2011) p62, Ref(2)P and ubiquitinated proteins are conserved markers of neuronal aging, aggregate formation and progressive autophagic defects. Autophagy 7: 572–583. 21325881 10.4161/auto.7.6.14943 PMC3127048

[27] Bera M, Kotamarthi HC, Dutta S, Ray A, Ghosh S, Bhattacharyya D, et al (2014) Characterization of Unfolding Mechanism of Human Lamin A Ig Fold by Single-Molecule Force Spectroscopy-Implications in EDMD. Biochemistry 53: 7242–7258. 10.1021/bi500726f 25343322

[28] Scharner J, Lu HC, Fraternali F, Ellis JA, Zammit PS (2014) Mapping disease-related missense mutations in the immunoglobulin-like fold domain of lamin A/C reveals novel genotype-phenotype associations for laminopathies. Proteins 82: 904–915. 10.1002/prot.24465 24375749

[29] Brown CA, Lanning RW, McKinney KQ, Salvino AR, Cherniske E, Crowe CA, et al (2001) Novel and recurrent mutations in lamin A/C in patients with Emery-Dreifuss muscular dystrophy. Am J Med Genet 102: 359–367. 11503164 10.1002/ajmg.1463

[30] Cao K, Blair CD, Faddah DA, Kieckhaefer JE, Olive M, Erdos MR, et al (2011) Progerin and telomere dysfunction collaborate to trigger cellular senescence in normal human fibroblasts. J Clin Invest 121: 2833–2844. 10.1172/JCI43578 21670498 PMC3223819

[31] Ostlund C, Bonne G, Schwartz K, Worman HJ (2001) Properties of lamin A mutants found in Emery-Dreifuss muscular dystrophy, cardiomyopathy and Dunnigan-type partial lipodystrophy. Journal of cell science 114: 4435–4445. 11792809 10.1242/jcs.114.24.4435

[32] Choi JC, Muchir A, Wu W, Iwata S, Homma S, Morrow JP, et al (2012) Temsirolimus activates autophagy and ameliorates cardiomyopathy caused by lamin A/C gene mutation. Science translational medicine 4: 144ra102 10.1126/scitranslmed.3003875 22837537 PMC3700376

[33] Magracheva E, Kozlov S, Stewart CL, Wlodawer A, Zdanov A (2009) Structure of the lamin A/C R482W mutant responsible for dominant familial partial lipodystrophy (FPLD). Acta crystallographica Section F, Structural biology and crystallization communications 65: 665–670. 10.1107/S1744309109020302 19574635 PMC2705630

[34] Burke B, Stewart CL (2006) The laminopathies: the functional architecture of the nucleus and its contribution to disease. Annu Rev Genomics Hum Genet 7: 369–405. 16824021 10.1146/annurev.genom.7.080505.115732

[35] Gotzmann J, Foisner R (2006) A-type lamin complexes and regenerative potential: a step towards understanding laminopathic diseases? Histochem Cell Biol 125: 33–41. 16142451 10.1007/s00418-005-0050-8

[36] Simon DN, Domaradzki T, Hofmann WA, Wilson KL (2013) Lamin A tail modification by SUMO1 is disrupted by familial partial lipodystrophy-causing mutations. Mol Biol Cell 24: 342–350. 10.1091/mbc.E12-07-0527 23243001 PMC3564541

[37] Christians ES, Benjamin IJ (2012) Proteostasis and REDOX state in the heart. Am J Physiol Heart Circ Physiol 302: H24–37. 10.1152/ajpheart.00903.2011 22003057 PMC3334238

[38] Christians ES, Mustafi SB, Benjamin IJ (2014) Chaperones and cardiac misfolding protein diseases. Curr Protein Pept Sci 15: 189–204. 24694370 10.2174/1389203715666140331111518

[39] Zhang X, Min X, Li C, Benjamin IJ, Qian B, Ding Z, et al (2010) Involvement of reductive stress in the cardiomyopathy in transgenic mice with cardiac-specific overexpression of heat shock protein 27. Hypertension 55: 1412–1417. 10.1161/HYPERTENSIONAHA.109.147066 20439823

[40] Verstraeten VL, Caputo S, van Steensel MA, Duband-Goulet I, Zinn-Justin S, Kamps M, et al (2009) The R439C mutation in LMNA causes lamin oligomerization and susceptibility to oxidative stress. J Cell Mol Med 13: 959–971. 10.1111/j.1582-4934.2009.00690.x 19220582 PMC3823411

[41] Badia MC, Giraldo E, Dasi F, Alonso D, Lainez JM, Lloret A, et al (2013) Reductive stress in young healthy individuals at risk of Alzheimer disease. Free Radic Biol Med 63: 274–279. 10.1016/j.freeradbiomed.2013.05.003 23665394

[42] Teodoro JS, Rolo AP, Palmeira CM (2013) The NAD ratio redox paradox: why does too much reductive power cause oxidative stress? Toxicol Mech Methods 23: 297–302. 10.3109/15376516.2012.759305 23256455

[43] Wilson KL, Foisner R (2010) Lamin-binding Proteins. Cold Spring Harbor perspectives in biology 2: a000554 10.1101/cshperspect.a000554 20452940 PMC2845209

[44] Singla A, Griggs NW, Kwan R, Snider NT, Maitra D, Ernst SA, et al (2013) Lamin aggregation is an early sensor of porphyria-induced liver injury. J Cell Sci 126: 3105–3112. 10.1242/jcs.123026 23641075 PMC3711202

[45] Arrasate M, Mitra S, Schweitzer ES, Segal MR, Finkbeiner S (2004) Inclusion body formation reduces levels of mutant huntingtin and the risk of neuronal death. Nature 431: 805–810. 15483602 10.1038/nature02998

[46] Ramos FJ, Chen SC, Garelick MG, Dai DF, Liao CY, Schreiber KH, et al (2012) Rapamycin reverses elevated mTORC1 signaling in lamin A/C-deficient mice, rescues cardiac and skeletal muscle function, and extends survival. Sci Transl Med 4: 144ra103 10.1126/scitranslmed.3003802 22837538 PMC3613228

[47] Park YE, Hayashi YK, Bonne G, Arimura T, Noguchi S, Nonaka I, et al (2009) Autophagic degradation of nuclear components in mammalian cells. Autophagy 5: 795–804. 19550147 10.4161/auto.8901

[48] Delaglio F, Grzesiek S, Vuister GW, Zhu G, Pfeifer J, Bax A (1995) NMRPipe: a multidimensional spectral processing system based on UNIX pipes. J Biomol NMR 6: 277–293. 8520220 10.1007/BF00197809

[49] Lee W, Westler WM, Bahrami A, Eghbalnia HR, Markley JL (2009) PINE-SPARKY: graphical interface for evaluating automated probabilistic peak assignments in protein NMR spectroscopy. Bioinformatics 25: 2085–2087. 10.1093/bioinformatics/btp345 19497931 PMC2723000

[50] Johnson BA (2004) Using NMRView to visualize and analyze the NMR spectra of macromolecules. Methods Mol Biol 278: 313–352. 15318002 10.1385/1-59259-809-9:313

[51] Shaffer CD, Wuller JM, Elgin SC (1994) Raising large quantities of Drosophila for biochemical experiments. Method Cell Biol 44: 99–108. 7707979 10.1016/s0091-679x(08)60908-5

[52] Schulze SR, Curio-Penny B, Li Y, Imani RA, Rydberg L, Geyer PK, et al (2005) Molecular genetic analysis of the nested *Drosophila melanogaster Lamin C* gene. Genetics 171: 185–196. 15965247 10.1534/genetics.105.043208 PMC1456510

[53] Lammerding J, Lee RT (2009) Mechanical properties of interphase nuclei probed by cellular strain application. Method Mol Biol 464: 13–26. 10.1007/978-1-60327-461-6_2 18951177 PMC4153730

[54] Downey T (2006) Analysis of a multifactor microarray study using Partek genomics solution. Method Enzymol 411: 256–270. 16939794 10.1016/S0076-6879(06)11013-7

[55] Pircs K, Nagy P, Varga A, Venkei Z, Erdi B, Hegedus K, et al (2012) Advantages and limitations of different p62-based assays for estimating autophagic activity in Drosophila. PLoS One 7: e44214 10.1371/journal.pone.0044214 22952930 PMC3432079

[56] Tietze F (1969) Enzymic method for quantitative determination of nanogram amounts of total and oxidized glutathione: applications to mammalian blood and other tissues. Anal Biochem 27: 502–522. 4388022 10.1016/0003-2697(69)90064-5

[57] Griffith OW (1980) Determination of glutathione and glutathione disulfide using glutathione reductase and 2-vinylpyridine. Anal Biochem 106: 207–212. 7416462 10.1016/0003-2697(80)90139-6

[58] Zhang Z, Yu J, Stanton RC (2000) A method for determination of pyridine nucleotides using a single extract. Anal Biochem 285: 163–167. 10998277 10.1006/abio.2000.4701

[59] Glock GE, Mc LP (1953) Further studies on the properties and assay of glucose 6-phosphate dehydrogenase and 6-phosphogluconate dehydrogenase of rat liver. Biochem J 55: 400–408. 13105646 10.1042/bj0550400 PMC1269290

[60] Rzezniczak TZ, Merritt TJ (2012) Interactions of NADP-reducing enzymes across varying environmental conditions: a model of biological complexity. G3 (Bethesda) 2: 1613–1623. 10.1534/g3.112.003715 23275884 PMC3516483

