# Supplementary figures and images for: Myopathic Lamin Mutations Cause Reductive Stress and Activate the Nrf2/Keap-1 Pathway
Source: PLoS Genet. 2015 May 21;11(5):e1005231. doi: 10.1371/journal.pgen.1005231 (PMC4440730; doi:10.1371/journal.pgen.1005231)

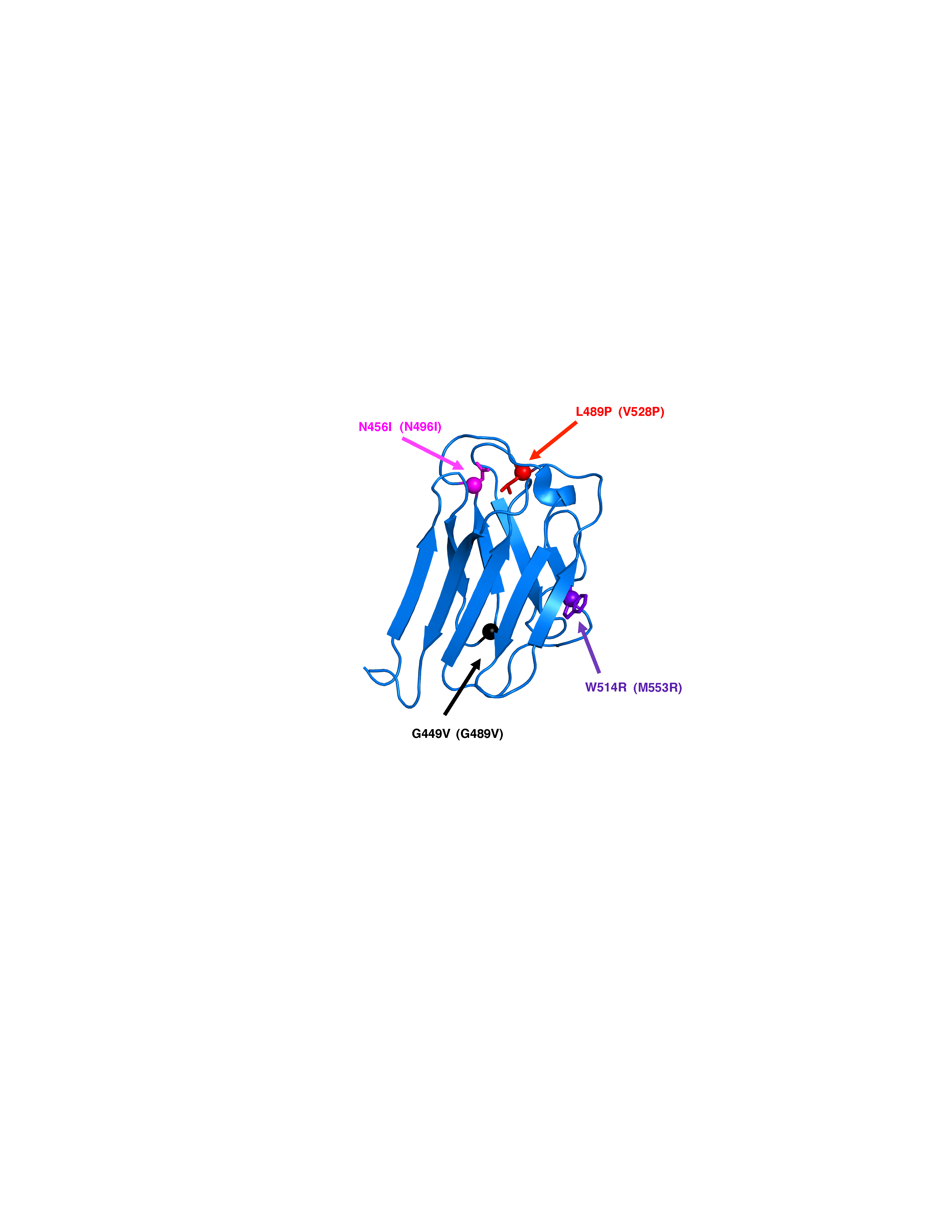

Supplement: S1 Fig — Ribbon plot of the Ig-fold domain of human lamin A/C (PDB 1IVT). Amino acid residues altered by mutations in the human LMNA gene are indicated. The corresponding amino acid substitutions in Drosophila Lamin C are indicated in parentheses. (TIF) [file pgen.1005231.s001.tif]

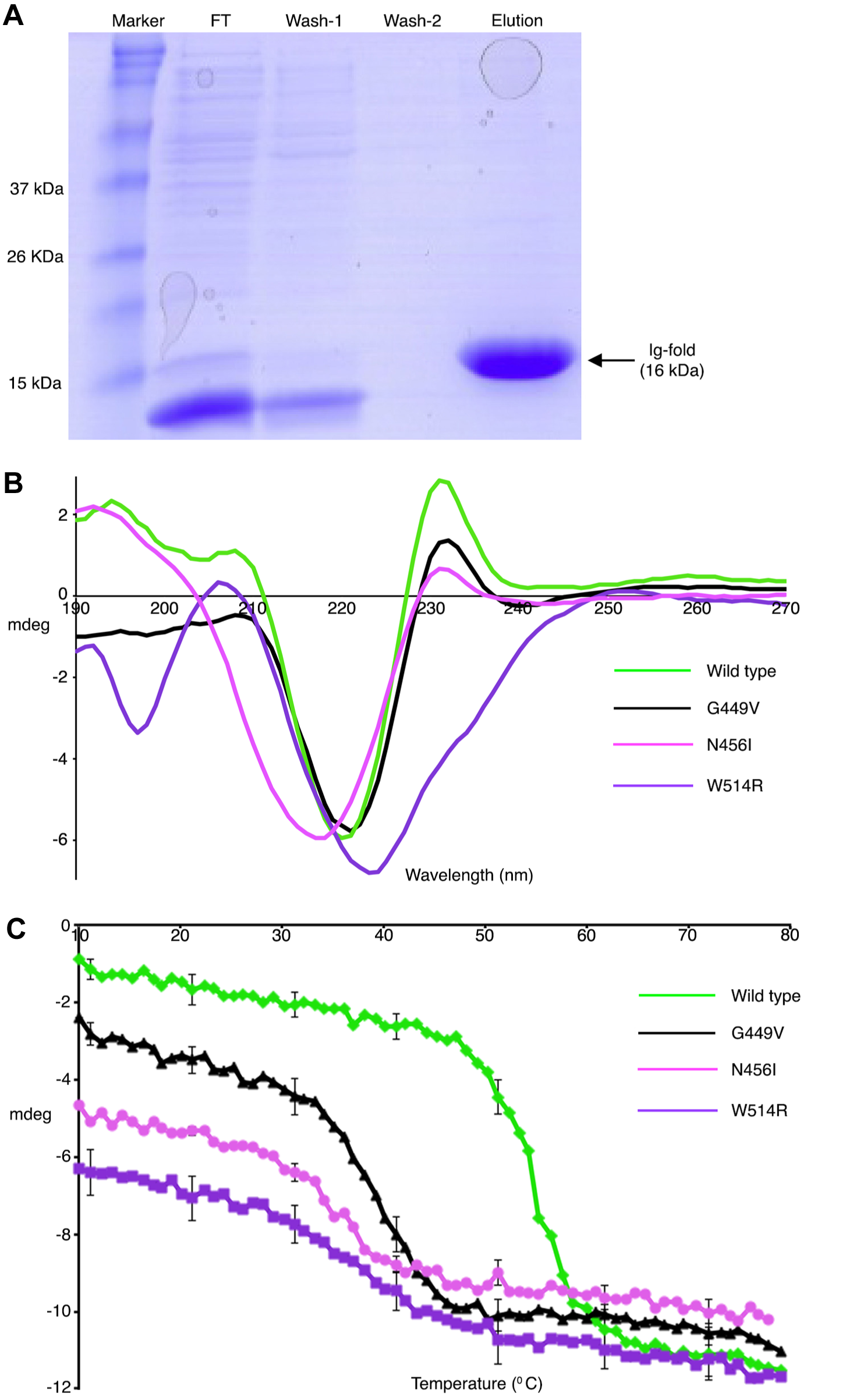

Supplement: S2 Fig — (A) Analysis of the purified wild type human lamin A/C Ig-fold domain using SDS PAGE following nickel column chromatography. Molecular weight markers are in lane 1. The flow through (FT), first wash (Wash-1), second wash (Wash-2) and elute (Elution) fractions are shown in lanes 2–5. The nickel affinity purified protein migrates to the anticipated molecular weight of the wild type Ig-fold, ~16 kDa. (B) CD spectra for the wild type and mutant Ig-fold domains expressed and purified from E. coli. The wild type and mutant Ig-fold domains possess beta sheet content as indicated by the peaks at 220 nm. The W514R substitution shows the absence of the peak at 232 nm due to the replacement of tryptophan. The majority of the L489P protein were unfolded as shown by the NMR data, therefore, CD data were not collected on this mutant. (C) Melting curves of wild type and mutant Ig-fold domains as determined by CD analysis at different temperatures. The T1/2, an indicator of thermal stability, was determined by calculating the midpoint of the curve between the start and end of the slope and are reported in the text. (TIF) [file pgen.1005231.s002.tif]

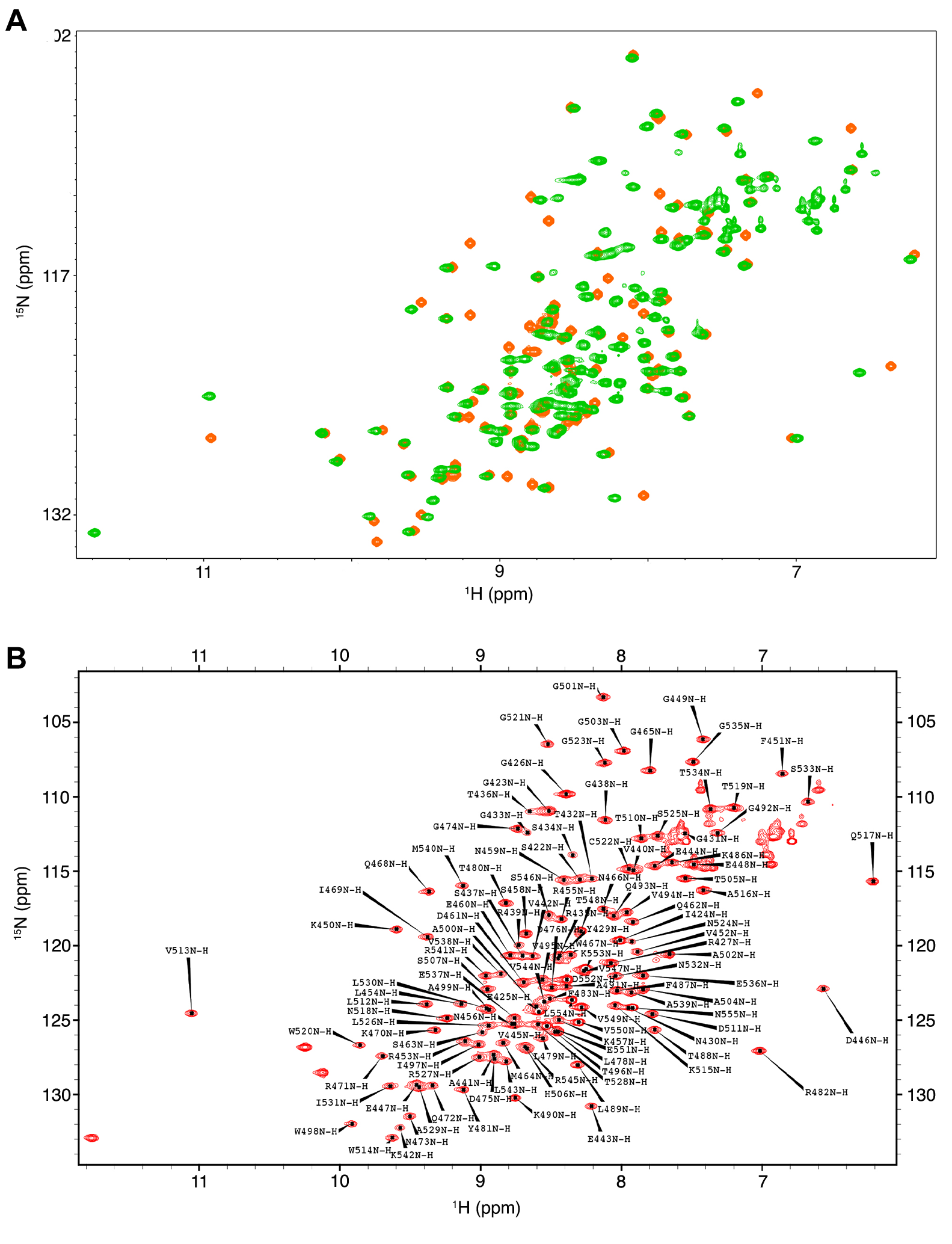

Supplement: S3 Fig — (A) Overlay of 15N/1H HSQC spectra of our Ig-fold domain construct (residues 435–552, green) and the reported Ig-fold construct (residues 428–549, reconstructed from BMRB5224, orange). The spectra are very similar indicating that they have similar tertiary structures. Subtle differences between the spectra are likely due to slight differences in the constructs used (see text). Due to these subtle differences, we performed backbone assignments of the amide cross peaks in order to unambiguously interpret the chemical shift perturbation data upon mutation. (B) Assigned 15N/1H HSQC spectrum of the Ig-fold domain of our wild type Ig-fold construct. The cross peaks were assigned by collecting and analyzing a suite of triple resonance NMR experiments using a 15N, 13C-labeled sample. (TIF) [file pgen.1005231.s003.tif]

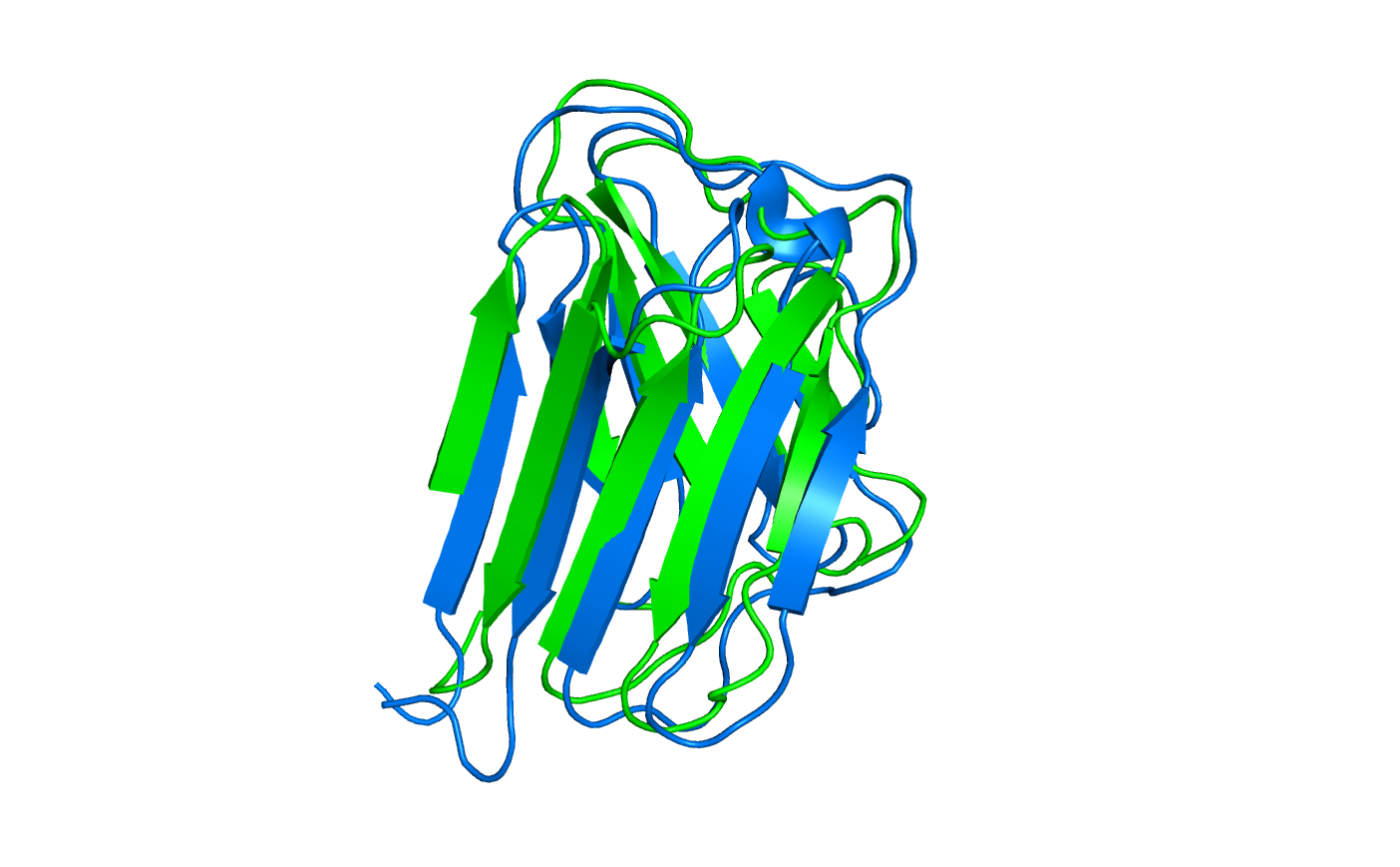

Supplement: S4 Fig — The protein fold prediction program (HHpred-homology) was used to generate a predicted structure for the Ig-fold sequence of Drosophila Lamin C (green). This predicted structure was compared to that of the known structure of the human lamin A/C Ig-fold (PDB 1IVT) (blue). (TIF) [file pgen.1005231.s004.tif]

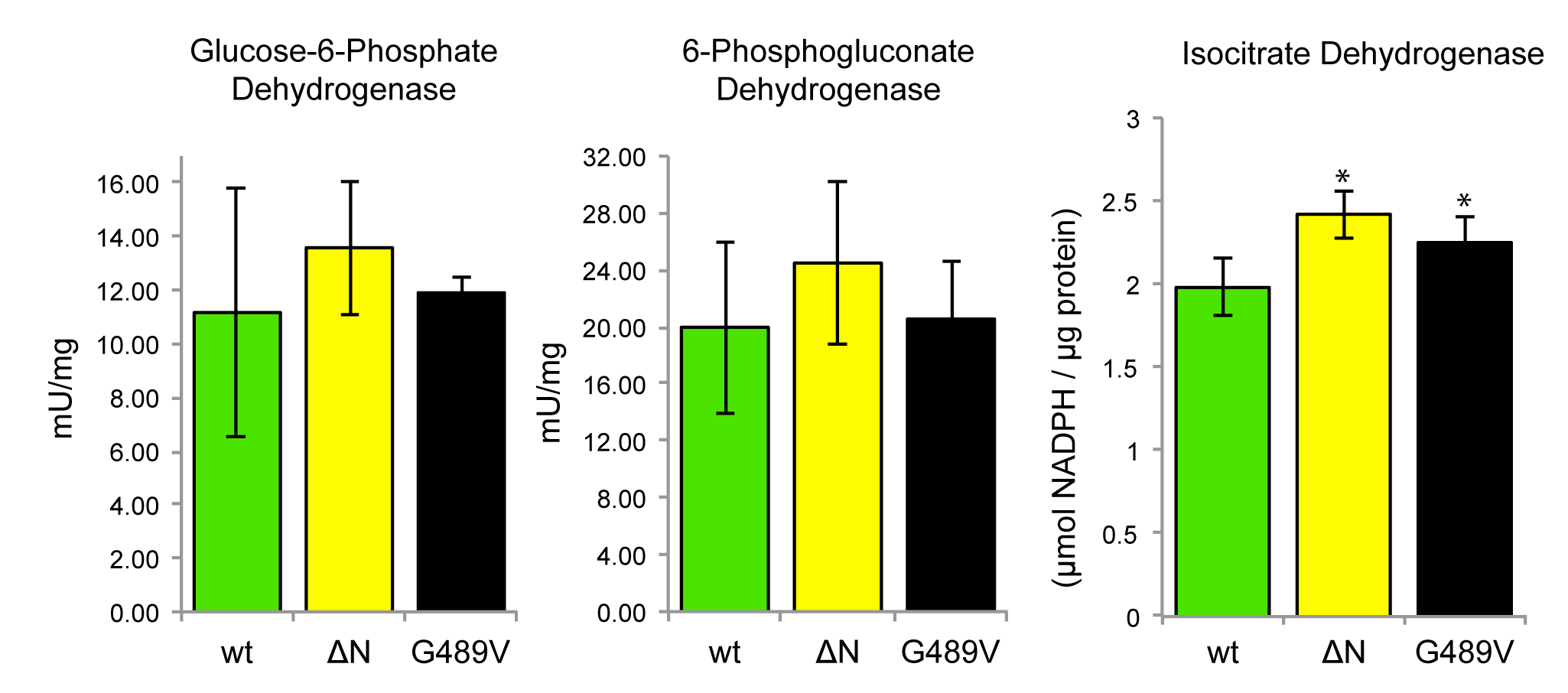

Supplement: S5 Fig — Quantitation of the activity of NADPH-producing enzymes, G6PD, 6PGD and IDH in Drosophila body wall muscles of larvae expressing mutant and wild type Lamin C. Analysis was performed on three independent biological samples. Error bars represent standard error of the mean. Statistical significance is indicated by * for p ≤ 0.05 when compared to values obtained for muscles expressing wild type Lamin C. (TIF) [file pgen.1005231.s005.tif]

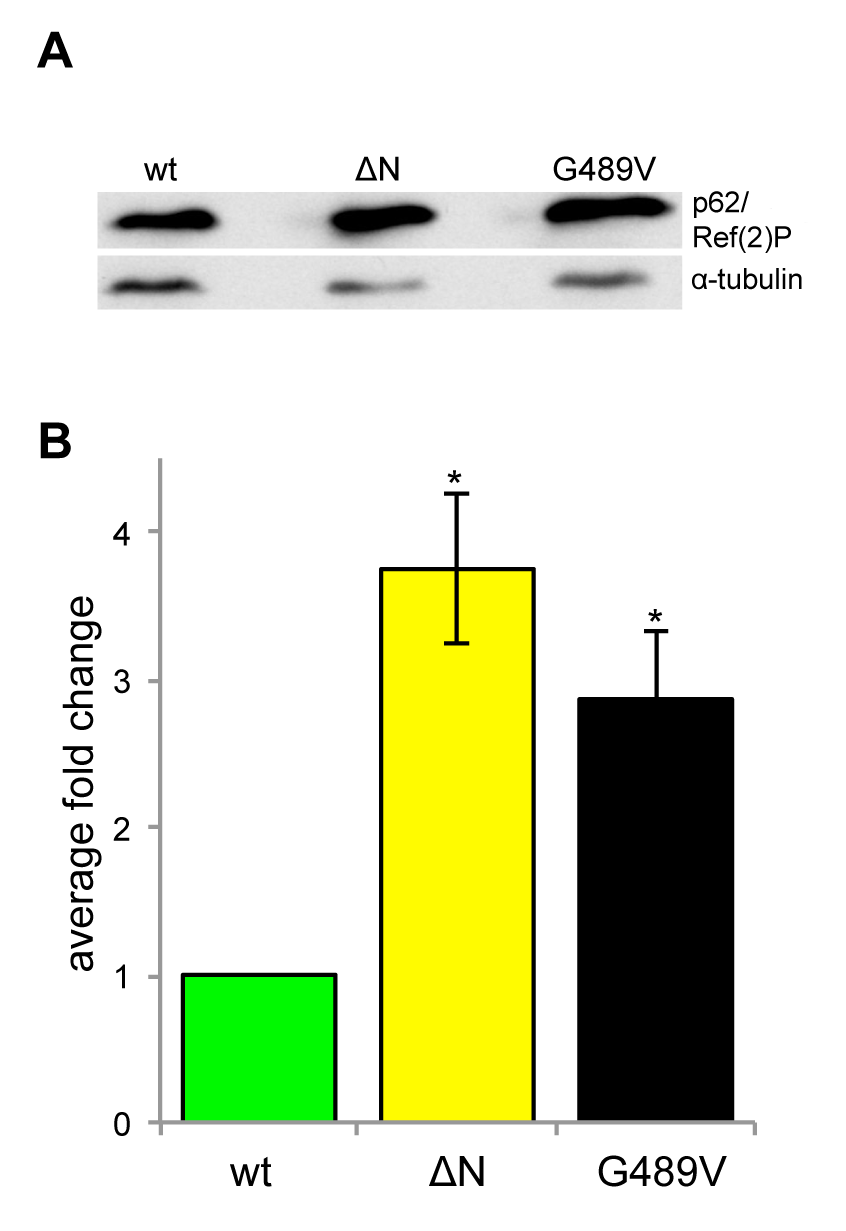

Supplement: S6 Fig — (A) Western analysis of total protein isolated from body wall muscle showing increased levels of p62 in larvae expressing mutant lamin relative to the wild type control. An antibody to alpha tubulin was used as a loading control. (B) Graphical representation of the data obtained three westerns performed on independent biological samples is shown below. Error bars represent standard error of the mean. The * indicates p ≤ 0.05 when compared to the value obtained for wild type Lamin C. (TIF) [file pgen.1005231.s006.tif]
