# Supplementary material for: Myopathic Lamin Mutations Cause Reductive Stress and Activate the Nrf2/Keap-1 Pathway
Source: PLoS Genet. 2015 May 21;11(5):e1005231. doi: 10.1371/journal.pgen.1005231 (PMC4440730; doi:10.1371/journal.pgen.1005231)
Supplement: S1 Table — Microarray analysis was performed using RNA isolated from muscle of third instar larvae expressing wild type Lamin C and Lamin C ΔN. The Partek software suite was used to identify genes that changes expression two-fold or greater with a p value of 0.05 or greater. (DOCX) [file pgen.1005231.s007.docx]

Table S1: Changes in gene expression between wild type Lamin C and ΔN

| **Gene symbol** | **Gene name** | **Fold change** | **p value** | **Function** |
| --- | --- | --- | --- | --- |
| *Cyp4p2* | *Cyp4p2* | 144.4 | 7.53E-07 | Electron carrier activity; heme binding; iron ion binding; oxidoreductase activity* |
| *PlexB* | *Plexin B* | 23.84 | 4.09E-08 | Axon guidance |
| *Ets65A* | *Ets at 65A* | 12.6 | 6.22E-08 | DNA binding; sequence-specific DNA binding; transcription factor activity* |
| *GstD5* | *Glutathione S transferase D5* | 10.47 | 1.27E-04 | Glutathione transferase activity |
| *CG9935* | *-* | 8.77 | 4.18E-06 | Extracellular-glutamate-gated ion channel activity* |
| *CG34007* | *-* | 7.96 | 7.33E-05 | Unknown |
| *CG1074* | *-* | 7.36 | 2.87E-07 | Methyltransferase activity; nucleic acid binding* |
| *CG31781* | *-* | 5.50 | 5.18E-06 | Lateral inhibition in cell fate determination |
| *GstD9* | *Glutathione S transferase D9* | 4.91 | 9.40E-05 | Glutathione transferase activity |
| *CG2064* | *-* | 4.21 | 1.43E-04 | Oxidoreductase activity* |
| *RluA-1* | *RluA-1* | 4.07 | 1.74E-04 | RNA binding; pseudouridine synthase activity; diaminohydroxyphosphoribosylaminopyrimidine deaminase activity* |
| *CG32021* | *-* | 3.39 | 3.25E-05 | Lateral inhibition in cell fate determination |
| *CG14906* | *-* | 3.72 | 4.37E-06 | Methyltransferase activity; nucleic acid binding* |
| *CG33494* | *-* | 3.60 | 9.57E-05 | Unknown |
| *Arc1* | *Activity-regulated cytoskeleton associated protein 1* | 2.89 | 4.82E-05 | Nucleic acid binding; zinc ion binding* |
| *CG3630* | *-* | 2.54 | 1.62E-05 | Unknown |
| *CG3448* | *-* | 2.41 | 5.93E-05 | DNA binding* |
| *alphaTub84D* | *alpha-Tubulin at 84D* | 2.39 | 7.62E-05 | GTP binding; GTPase binding; structural constituent of cytoskeleton*; Myosin binding |
| *CG33205* | *-* | 2.06 | 1.46E-04 | Mesoderm development |
| *RpS11* | *Ribosomal protein S11* | 2.04 | 2.74E-05 | Structural constituent of ribosome* |
| *CG16787* | *-* | 2.03 | 2.18E-05 | Unknown |
| *Plum* | *Plum* | -2.11 | 2.01E-05 | Negative regulator of synaptic growth at the neuromuscular junction; neuron remodeling |
| *Sclp* | *Sclp* | -2.49 | 7.23E-06 | Muscle function |
| *CG10365* | *-* | -2.54 | 1.77E-04 | Unknown |
| *NnaD* | *Nna1 ortholog* | -2.56 | 4.34E-05 | Metallocarboxypeptidase activity; purine nucleotide binding; zinc binding*; larval and neural retina development; mitochondrion organization |
| *l(2)03659* | *lethal(2)03659* | -5.08 | 1.47E-06 | ATP binding; ATPase activity; couple to transmembrane movement of substances; transporter activity* |
| *gkt* | *glaikit* | -6.31 | 1.60E-07 | 3’-tyrosyl-DNA phosphodiesterase activity*; central nervous system development; establishment and maintenance of epithelial cell apical/basil polarity |
| *CG32850* | *-* | -6.98 | 1.82E-05 | Ubiquitin-protein transferase activity; zinc ion binding* |

*Inferred from amino acid sequence
