# Supplementary material for: Myopathic Lamin Mutations Cause Reductive Stress and Activate the Nrf2/Keap-1 Pathway
Source: PLoS Genet. 2015 May 21;11(5):e1005231. doi: 10.1371/journal.pgen.1005231 (PMC4440730; doi:10.1371/journal.pgen.1005231)
Supplement: S2 Table — Microarray analysis was performed using RNA isolated from muscle of third instar larvae expressing wild type Lamin C and Lamin C G489V. The Partek software suite was used to identify genes that changes expression two-fold or greater with a p value of 0.05 or greater. (DOCX) [file pgen.1005231.s008.docx]

Table S2: Changes in gene expression between wild type Lamin C and G489V

| **Gene symbol** | **Gene title** | **Fold change** | **p value** | **Function** |
| --- | --- | --- | --- | --- |
| *Cyp4p2* | *Cyp4p2* | 71.70 | 1.85E-06 | Electron carrier activity; heme binding; iron binding; oxidreductase activity* |
| *Cyp6a17* | *Cytochrome P450-6a17* | 39.03 | 2.09E-05 | Electron carrier activity; heme binding; iron binding; oxidoreductase activity* |
| *PlexB* | *Plexin B* | 34.56 | 2.11E-08 | Axon guidance |
| *CG34007* | *-* | 33.49 | 3.47E-06 | Unknown |
| *Takr99D* | *Tachykinin-like receptor at 99D* | 20.44 | 1.46E-06 | Neuropeptide receptor activity*; tachykinin receptor activity |
| *CG34115* | *-* | 16.52 | 6.57E-05 | Unknown |
| *NfI* | *Nuclear factor I* | 14.87 | 2.34E-04 | Sequence-specific DNA binding transcription factor activity; DNA replication and transcription* |
| *Prx2540-2* | *Peroxiredoxin 2540-2* | 13.71 | 3.97E-05 | Glutathione peroxidase activity*; thioredoxin peroxidase activity |
| *GstD9* | *Glutathione S transferase D9* | 8.66 | 1.63E-05 | Glutathione transferase activity |
| *pan* | *pangolin* | 7.48 | 1.32E-05 | Beta-catenin binding*; kinase binding; sequence specific DNA binding transcription factor |
| *GstD4* | *Glutathione S transferase D4* | 7.13 | 1.97E-05 | Glutathione transferase activity |
| *CG7130* | *-* | 6.84 | 8.33E-05 | Unknown |
| *CG14866* | *-* | 6.64 | 2.05E-05 | Carbohydrate binding* |
| *CG5955* | *-* | 6.60 | 3.11E-05 | Coenzyme binding; UDP-glucose 4-epimerase activity* |
| *CG3448* | *-* | 6.14 | 8.68E-07 | DNA binding* |
| *CG2064* | *-* | 5.88 | 4.35E-05 | Oxidoreductase activity* |
| *CG2065* | *-* | 5.64 | 4.53E-04 | Oxidoreductase activity* |
| *CG3008* | *-* | 5.46 | 2.08E-04 | ATP binding; protein kinase activity; protein serine/threonine kinase activity* |
| *Ilp5* | *Insulin-like peptide 5* | 5.12 | 2.01E-04 | Hormone activity; insulin receptor binding* |
| *CG14673* | *-* | 4.46 | 1.21E-04 | Unknown |
| *Dph5* | *Diphthamide methylatransferase* | 4.38 | 2.66E-05 | Diphthinine synthesis activity* |
| *CG14906* | *-* | 4.34 | 2.27E-06 | Methyltransferase activity; nucleic acid binding* |
| *CG31781* | *-* | 4.21 | 3.05E-04 | Lateral inhibition in cell fate determination |
| *Nxt1* | *NTF2-related export protein 1* | 3.97 | 3.81E-04 | Unknown |
| *Fos28F* | *Fos-related gene at 28F* | 3.82 | 9.44E-06 | Mechanical gated ion channel activity; mechanosensory behavior |
| *CG14451* | *-* | 3.80 | 1.67E-04 | Metal ion binding* |
| *gp210* | *gp210* | 3.80 | 2.70E-05 | Nuclear transport |
| *CG9935* | *-* | 3.48 | 1.03E-05 | Glutamate gated ion channel activity* |
| *CG33494* | *-* | 3.42 | 1.21E-04 | Unknown |
| *CG12868* | *-* | 3.42 | 1.60E-04 | Unknown |
| *Nmnat* | *Nicotinamide mononucleotide adenylyltransferase 1* | 3.39 | 2.97E-04 | Nicotinamide nucleotide adenylyltransferase activity; unfolded protein binding |
| *CG31953* | *-* | 3.38 | 1.95E-05 | Unknown |
| *CG2076* | *-* | 3.32 | 2.09E-04 | Unknown |
| *CG18673* | *-* | 3.18 | 2.65E-04 | Carbonate dehydratase activity* |
| *Arc1* | *Activity-regulated cytoskeleton associated protein 1* | 3.07 | 3.47E-05 | Nucleic acid binding; zinc binding* |
| *CG16787* | *-* | 3.01 | 1.48E-06 | Unknown |
| *CG14655* | *-* | 3.05 | 4.12E-04 | Metal ion binding; nucleic acid binding* |
| *Gr63α* | *Gustatory receptor 63α* | 3.03 | 4.26E-04 | Taste receptor activity* |
| *CG42575* | *-* | 3.01 | 4.40E-04 | Sodium dependent phosphate transmembrane transporter activity |
| *CG12728* | *-* | 3.00 | 3.38E-04 | DNA repair* |
| *bocksbeutel* | *bocksbeutel* | 2.76 | 9.99E-05 | LEM domain inner nuclear envelope protein |
| *dpr7* | *dpr7* | 2.71 | 3.94E-05 | Sensory perception of chemical stimulus* |
| *CG32021* | *-* | 2.68 | 2.11E-04 | Lateral inhibition |
| *alphaTub84D* | *alpha-Tubulin at 84B* | 2.66 | 3.93E-05 | GTP binding; ATPase activity; structural constituent of cytoskeleton*; myosin binding |
| *Prp31* | *-* | 2.62 | 9.40E-05 | Spliceosome assembly; mRNA splicing*; neurogenesis |
| *Mef2* | *Myocyte enhancer factor 2* | 2.58 | 5.12E-05 | Sequence specific DNA binding transcription factor; muscle development |
| *Irbp* | *Inverted repeat binding protein* | 2.58 | 2.72E-04 | ATP-dependent DNA helicase activity; damaged DNA binding; DNA dependent protein kinase activity* |
| *Ada2b* | *Transcriptional adapter 2S* | 2.54 | 2.92E-04 | Histone acetyltransferase activity*; chromatin remodeling |
| *CG6171* | *-* | 2.47 | 4.17E-06 | 3’-5’ exonuclease activity; DNA lyase activity; endodeoxyribonuclease activity |
| *CG14164* | *-* | 2.41 | 3.45E-04 | Unknown |
| *unc-13* | *unc-13* | 2.38 | 2.82E-04 | Calmodulin binding; diacylglycerol binding* |
| *CG5961* | *-* | 2.36 | 1.04E-04 | Ubiquitin transferase activity* |
| *Cpr66D* | *Cuticular protein 66D* | 2.36 | 4.28E-04 | Structural constituent of chitin based cuticle* |
| *CG7135* | *-* | 2.35 | 2.55E-04 | Transfer phosphorus groups* |
| *CG32000* | *-* | 2.31 | 1.71E-04 | ATPase activity, transmembrane movement of ions; metal ion binding; nucleotide binding* |
| *CG9945* | *-* | 2.30 | 7.37E-05 | Unknown |
| *CG10463* | *-* | 2.27 | 1.50E-04 | Flavin adenine dinucleotide binding; metal ion binding; tRNA dihydrouridine synthase activity* |
| *CG10638* | *-* | 2.26 | 6.33E-06 | Alditol:NADP+ 1-oxidoreductase activity* |
| *RpA-70* | *Replication Protein A 70* | 2.21 | 4.06E-04 | Single-stranded DNA binding; DNA replication and repair |
| *Fem-1* | *Fem-1* | 2.18 | 3.15E-04 | Ubiquitin transferase activity* |
| *CG9836* | *-* | 2.17 | 1.78E-05 | Unknown |
| *membrin* | *membrin* | 2.14 | 4.61E-06 | SNAP-receptor activity* |
| *CG1074* | *-* | 2.14 | 8.15E-05 | Methyltransferase activity; nucleic acid binding* |
| *RpS11* | *Ribosomal protein S11* | 2.01 | 3.06E-05 | Structural constituent of ribosome* |
| *mGluR* | *metabotropic glutamate receptor* | 2.01 | 8.61E-05 | G-protein couple receptor activity*; cholesterol binding; glutamate binding |
| *CG9393* | *-* | -2.04 | 4.81E-05 | Glutathione transferase activity |
| *CG31817* | *-* | -2.04 | 4.18E-05 | Unknown |
| *CG8888* | *-* | -2.05 | 2.13E-04 | Oxidoreductase activity |
| *CG31436* | *-* | -2.15 | 2.49E-05 | Transferase activity, transferring phosphorus-containing groups* |
| *NnaD* | *Nna1 ortholog* | -2.46 | 5.55E-05 | Metallocarboxypeptidase activity; purine nucleotide binding; zinc binding*; larval and neural retina development; mitochondrion organization |
| *CG34349* | *-* | -2.58 | 1.07E-05 | Unknown |
| *Ho* | *Heme oxygenase* | -2.58 | 2.90E-05 | Heme oxygenase activity |
| *CG11475* | *-* | -2.61 | 1.03E-05 | Unknown |
| *l(2)efl* | *lethal(2) essential for life* | -2.79 | 1.78E-05 | Protein lipidation; regulation of autophagy; regulation of translation |
| *CG5171* | *-* | -3.61 | 4.59E-04 | Trehalose phosphate activity* |
| *AR-2* | *Allatostatin A Receptor 2* | -3.82 | 3.42E-04 | G-protein couple receptor activity; neuropeptide receptor activity*; allatostatin receptor activity |
| *Sh* | *Shaker* | -3.83 | 6.65E-05 | Potassium channel activity*; voltage-gated cation channel activity |
| *Sclp* | *-* | -3.88 | 7.11E-07 | Muscle function |
| *CG10365* | *-* | -4.20 | 1.51E-05 | Unknown |
| *CG13907* | *-* | -4.24 | 1.72E-04 | Secondary active monocarboxylate transmembrane transporter activity* |
| *l(2)03659* | *lethal(2)03659* | -4.60 | 2.13E-06 | ATP binding; ATPase activity; transporter activity* |
| *gkt* | *Glaikit* | -5.04 | 3.46E-07 | 3’-tyrosyl-DNA phosphodiesterase activity*; nervous system development; epithelial cell apical/basil polarity |
| *CG32850* | *-* | -5.47 | 3.94E-05 | Ubiquitin-protein transferase activity; zinc ion binding* |
| *CG2082* | *-* | -6.11 | 1.22E-04 | Unknown |
| *CG33143* | *-* | -6.94 | 2.31E-04 | Unknown |
| *CG42319* | *-* | -8.61 | 2.77E-04 | Unknown |
| *CG14022* | ***-*** | -13.22 | 3.50E-04 | Acylphosphatase activity* |

*Inferred from amino acid sequence
