# Supplementary material for: Myopathic Lamin Mutations Cause Reductive Stress and Activate the Nrf2/Keap-1 Pathway
Source: PLoS Genet. 2015 May 21;11(5):e1005231. doi: 10.1371/journal.pgen.1005231 (PMC4440730; doi:10.1371/journal.pgen.1005231)
Supplement: S3 Table — Primers were used to perform site-directed mutagenesis on a plasmid containing wild type Lamin C. (DOCX) [file pgen.1005231.s009.docx]

Table S3: Lamin C mutagenesis primers

| G449V forward | 5'-GAGGAGGTGGATGAGGAGGTCAAGTTTGTCC GGCTGCGC-3' |
| --- | --- |
| G449V reverse | 5'-GCGCAGCCGGACAAACTTGACCTCCTCATCCA CCTCCTC-3' |
| N456I forward | 5’–CAAGTTTGTCCGGCTGCGCATTAAGTCCAATG AGGACCAG-3' |
| N456I reverse | 5’–CTGGTCCTCATTGGACTTAATGCGCAGCCGGA CAAACTTG-3' |
| L489P forward | 5’–TTCCCACCAAAGTTCACCCCGAAGGCTGGGCA GGTGGTG-3' |
| L489P reverse | 5’–CACCACCTGCCCAGCCTTCGGGGTGAACTTTG  GTGGGAA-3' |
| W514R forward | 5’–CCCCCTACCGACCTGGTGCGCAAGGCACAGA  ACACCTGG-3' |
| W514R reverse | 5’-CCAGGTGTTCTGTGCCTTGCGCACCAGGTCGGTAGG GGG-3' |
